# Citizen science for monitoring the health and well-being related Sustainable Development Goals and the World Health Organization’s Triple Billion Targets

**DOI:** 10.3389/fpubh.2023.1202188

**Published:** 2023-08-09

**Authors:** Dilek Fraisl, Linda See, Diana Estevez, Nola Tomaska, Steve MacFeely

**Affiliations:** ^1^International Institute for Applied Systems Analysis, Laxenburg, Austria; ^2^World Health Organization, Geneva, Switzerland; ^3^University College Cork, Cork, Ireland

**Keywords:** Sustainable Development Goals (SDGs), Triple Billion Targets, data gaps and needs, official statistics, citizen science, health and well-being, health and well-being indicators

## Abstract

Achieving the health and well-being related Sustainable Development Goals (SDGs) and the World Health Organization’s (WHO) Triple Billion Targets depends on informed decisions that are based on concerted data collection and monitoring efforts. Even though data availability has been increasing in recent years, significant gaps still remain for routine surveillance to guide policies and actions. The COVID-19 crisis has shown that more and better data and strengthened health information systems are needed to inform timely decisions that save lives. Traditional sources of data such as nationally representative surveys are not adequate for addressing this challenge alone. Additionally, the funding required to measure all health and well-being related SDG indicators and Triple Billion Targets using only traditional sources of data is a challenge to achieving efficient, timely and reliable monitoring systems. Citizen science, public participation in scientific research and knowledge production, can contribute to addressing some of these data gaps efficiently and sustainably when designed well, and ultimately, could contribute to the achievement of the health and well-being related SDGs and Triple Billion Targets. Through a systematic review of health and well-being related indicators, as well as citizen science initiatives, this paper aims to explore the potential of citizen science for monitoring health and well-being and for mobilizing action toward the achievement of health and well-being related targets as outlined in the SDG framework and Triple Billion Targets. The results demonstrate that out of 58 health and well-being related indicators of the SDGs and Triple Billion Targets covered in this study, citizen science could potentially contribute to monitoring 48 of these indicators and their targets, mostly at a local and community level, which can then be upscaled at a national level with the projection to reach global level monitoring and implementation. To integrate citizen science with official health and well-being statistics, the main recommendation is to build trusted partnerships with key stakeholders including National Statistical Offices, governments, academia and the custodian agencies, which is mostly the WHO for these health and well-being related targets and indicators.

## Highlights

- Achieving the health and well-being related SDGs and the WHO’s Triple Billion Targets depends on better data and strengthened health information systems.
- Citizen science can increase the availability, quality, granularity, applicability and timeliness of health and well-being related data.
- 48 out of 58 health and well-being related indicators (83%) of the SDGs and the WHO’s Triple Billion Targets can benefit from citizen science data in a direct or supplementary way.
- Integrating citizen science with official health and well-being statistics requires trusted partnerships with key stakeholders, such as the National Statistical Offices, the custodian agencies and the citizen science community.

## 1. Introduction

The United Nations Sustainable Development Goals (UN SDGs), which were ratified in September 2015 by the member states of the UN, aim to end poverty, achieve prosperity and protect the planet, while leaving no one behind (1). Comprised of 17 overarching goals, and 169 targets, the SDGs are monitored through 231 unique indicators forming the Global Indicator Framework (GIF), which have been developed under the aegis of the Inter-agency and Expert Group on SDG Indicators (IAEG-SDGs) (2). One or more custodian agencies, which are typically UN agencies or other international organizations, have been assigned to each indicator for methodology development, data compilation and reporting at a global level (3). Custodian agencies are also responsible for developing techniques for filling data gaps and to ensure that indicators are comparable globally. When an indicator has an agreed methodology and good data coverage (defined as *the availability of data for at least 50% of countries and of the population in every region where the indicator is relevant*), it is classified as Tier I. Tier II refers to indicators where data are lacking. As of 30 November 2022, 148 indicators are Tier I, 77 indicators are Tier II and there are 6 indicators with components that have multiple tiers (4).

The primary responsibility for SDG monitoring lies with national governments, which is undertaken by National Statistical Offices (NSOs) and other relevant line ministries (2), often using traditional forms of data collection, such as censuses or household surveys, and administrative or routine monitoring and surveillance systems. The data provided at national level feed the Global SDG Indicators Data Platform, which are used to analyze progress on a regional and global scale (5). However, measuring all relevant indicators at national level comes at a considerable cost, and this represents a barrier to assessing progress made toward the SDGs, especially in the case of low- and lower-middle income countries (6). Moreover, as outlined above, about 33% of indicators are still classified as Tier II in 2023, signifying the persistence of large gaps in data availability, and even for Tier 1 indicators, only 19% of the required data are available to track progress on the SDGs across countries and over time (7).

In addition to the SDGs, the Triple Billion Targets, which are the basis of the WHO’s Thirteenth General Program of Work (GPW13) as a measurement and policy framework, also require timely, accurate and actionable data. The GPW13 was initially intended to cover the period 2019–2023, and then extended to 2025 (8). Providing strategic direction to WHO’s work, the GPW13 also serves as the basis for resource allocation and mobilization to achieve WHO’s mission, which is to “promote health, keep the world safe and serve the vulnerable.” GPW13 is structured based on three strategic priorities, namely, (i) to ensure healthy lives and well-being for all at all ages, (ii) achieving universal health coverage, and (iii) addressing health emergencies and promoting healthier populations. More specifically, these strategic priorities also constitute the Triple Billion Targets, which are (i) *one billion more people benefitting from universal health coverage, (ii) one billion more people better protected from health emergencies and (iii) one billion more people enjoying better health and well-being*. These targets have a particular focus on measuring impact, prioritizing the reduction of health inequalities, driving health impact in every country through leadership, resource mobilization, urgent action and building partnerships. The Triple Billion Targets and the GPW13 are based on the SDGs, as they align with them and thus can help countries deliver on the SDGs simultaneously. They include 46 indicators, 39 of which are already SDG indicators, such as the coverage of essential health services (SDG indicator 3.8.1) and safely managed drinking water (SDG indicator 6.1.1), among others. Seven additional indicators cover areas such as noncommunicable diseases (hypertension, obesity, trans fats policy), polio, antimicrobial resistance and health emergencies (vaccine coverage for epidemic prone diseases, provision of essential services to vulnerable populations) (9).

Both the health and well-being related SDGs and the Triple Billion Targets require strong health and well-being monitoring systems, which currently is not the case in many parts of the world. Health and well-being are challenging to define as they have different meanings in different socio-cultural contexts (10, 11). The WHO (12) defines health as “a state of complete physical, mental and social well-being, and not merely the absence of disease or infirmity.” This definition emphasizes well-being linking it to positive emotions and state (10, 13). Well-being has two conceptual dimensions as objective well-being and subjective well-being (14–16). Objective well-being focuses on aspects related to material and social attributes such as income, housing and education (17, 18), while subjective well-being refers to one’s own perception about their life and their level of satisfaction with it (15, 19, 20). In the context of this research, we consider health and well-being as defined in the SDGs, and more specifically in the targets of SDG 3 “Good Health and Well-being,” covering topics from reducing maternal mortality (Target 3.1) to achieving universal health coverage (Target 3.8). Beyond SDG 3, our definition also includes topics related to public, global and environmental health and covers issues such as violence against women, air quality or access to safely managed drinking water, among others, covered in different SDGs and Triple Billion Targets.

The challenging situation on data availability has been further compounded by the COVID-19 pandemic. Even though the pandemic is not explicitly covered by the SDGs or Triple Billion Targets, as it started after the adoption of both frameworks, it is highly relevant to both of them, as it has negatively affected the monitoring and achievement of many health and well-being related indicators and targets. For example, the 2022 Global SDG Report highlights that COVID-19 disrupted essential health services and posed major threats to societies beyond the risk to health (21). It has had an impact on children’s learning and well-being, and women have suffered from loss of jobs and livelihoods. It has also intensified inequalities within and among countries. The poorest and most vulnerable are the worst affected by the economic and social consequences of the virus (22). Additionally, inequities in vaccine distribution and health services are also a threat to improving people’s well-being more generally. As of May 2022, only 17% of people in low-income countries had received at least one dose of a vaccine, while in high-income countries, this proportion is more than 80% (23).

COVID-19 has also demonstrated that weak data and information systems pose an additional challenge to decision makers who need evidence to make timely decisions for interventions that could save lives. For example, only 38% of countries had the required monthly mortality data from January 2020 to December 2021 to understand COVID-19-related excess mortality, and a recent study by the WHO shows that excess deaths due to COVID-19 were 2.74 times greater than the 5.42 million reported for this period (24).

**Figure 1:**
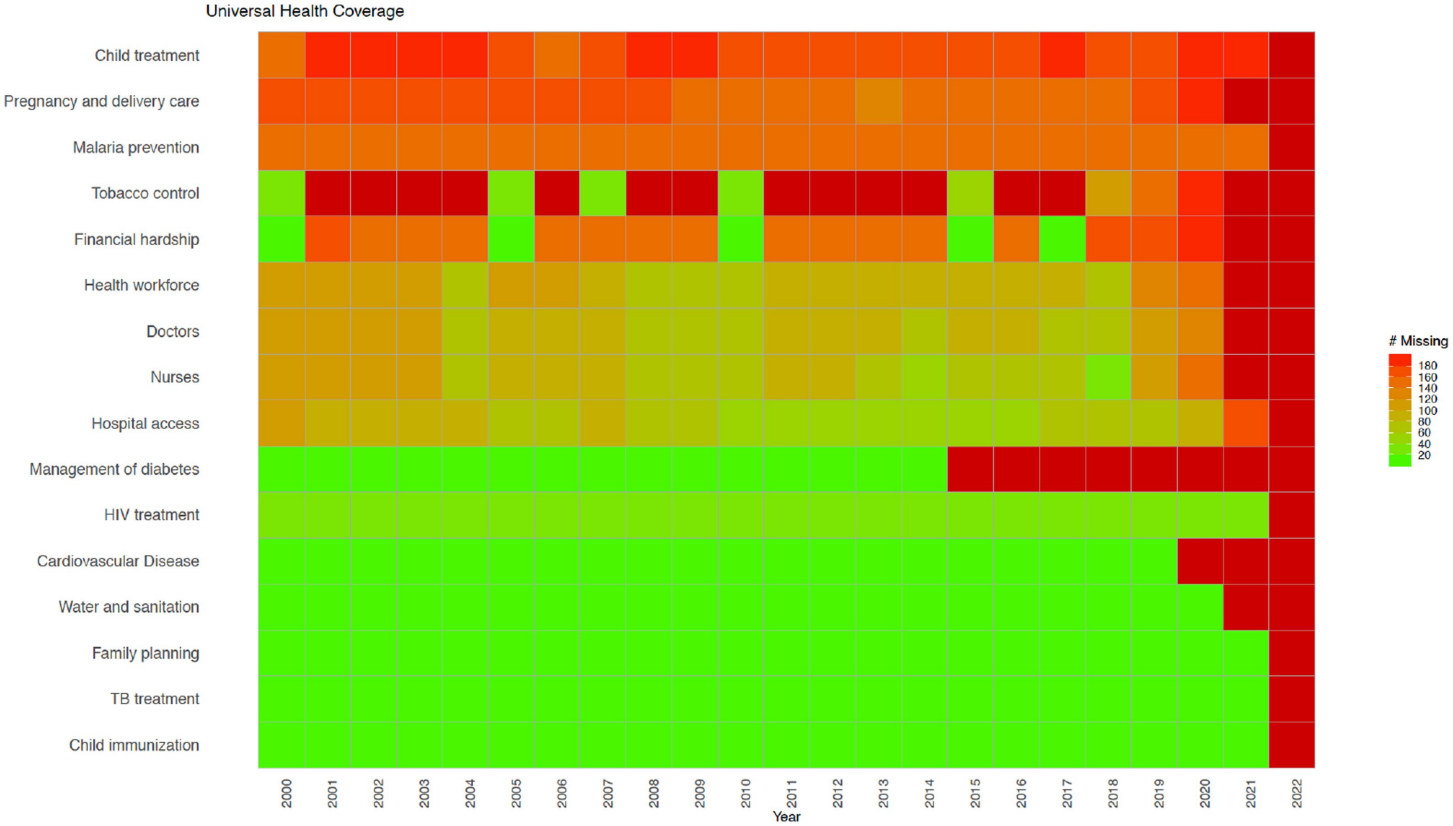
Universal Health Coverage indicators from the WHO’s Triple Billion Targets and the SDG framework. Dark red indicates that data are missing for all countries for the mentioned year while bright green on the opposite end of the spectrum shows that there are no missing values reported for all countries, which is 194 in total. Shades in between show partial data availability. Source: WHO, 2023.

**Figure 2:**
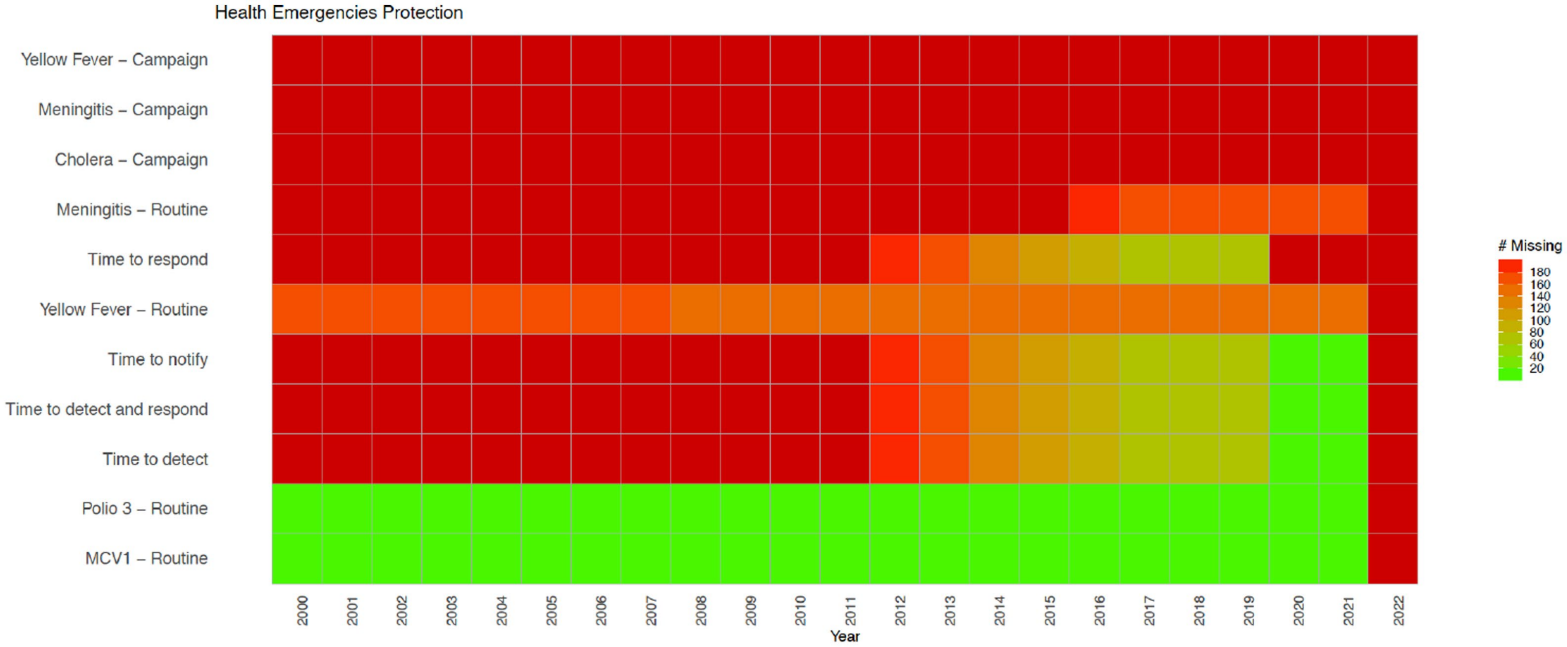
Health Emergencies indicators from the WHO’s Triple Billion Targets and the SDG framework. Dark red indicates that data are missing for all countries for the mentioned year while bright green on the opposite end of the spectrum shows that there are no missing values reported for all countries, which is 194 in total. Shades in between show partial data availability. Source: WHO, 2023.

**Figure 3:**
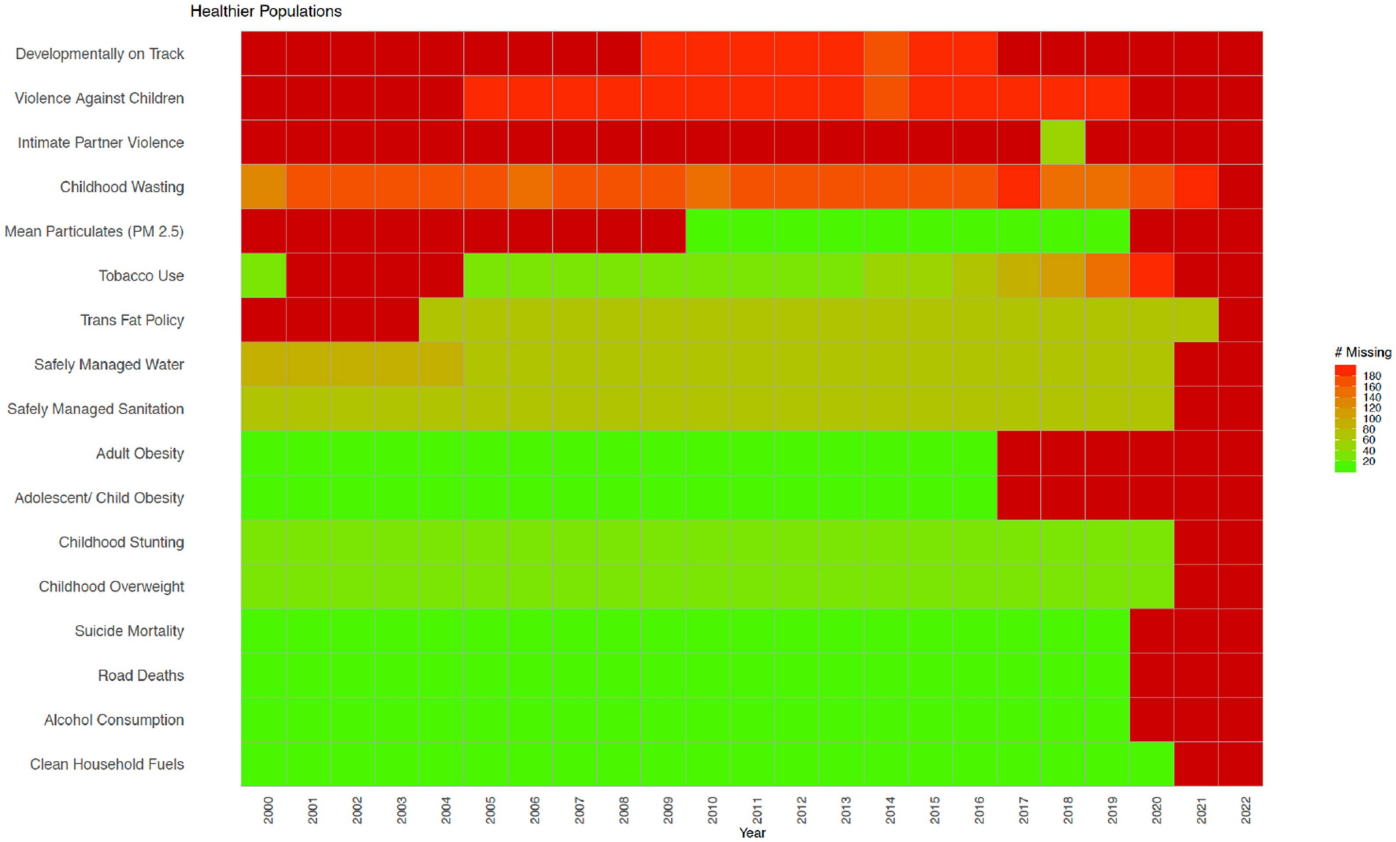
Healthier Populations indicators from the WHO’s Triple Billion Targets and the SDG framework. Dark red indicates that data are missing for all countries for the mentioned year while bright green on the opposite end of the spectrum shows that there are no missing values reported for all countries, which is 194 in total. Shades in between show partial data availability. Source: WHO, 2023.

Figures 1–3 show data availability for WHO’s Triple Billion Targets related to Universal Health Coverage, Health Emergencies Protection and Healthier Populations, which also include the health and well-being related SDG indicators covered in this study. These figures show that a large amount of data are missing. The situation related to data availability has not improved dramatically over the past 20 years, and much of the data, even when they exist, are not very timely. For example, in the Healthier Populations target (Figure 3), data related to most of the indicators are missing during or since COVID.

One concrete example on data availability is the *proportion of women (aged 15–49) having need for family planning satisfied with modern methods (%)*, which is both an SDG (3.7.1) and a Triple Billion Target indicator; 65 countries had no data available between 2000–2018, 54 countries have at least one data point available since 2015 or later, and 52 countries have trend data, which means they have at least two data points available, with the latest data from 2015 or later (9).

To address the challenges on data availability, alternative data sources and methods could be utilized for monitoring health and well-being (26, 27). Citizen science has recently been recognized as a potential source of data for the SDGs, e.g., to provide data at a higher spatial and temporal resolution or more detailed information than traditional, authoritative data sources alone might provide (6, 28, 29).

Citizen science is broadly defined as the public involvement in scientific research and knowledge production (30). Monitoring birds, measuring water quality, examining satellite imagery for missing airplanes, reporting on sexual or physical violence, collecting litter and data from beaches and riverbanks are all examples of citizen science. Citizen science includes diverse methodologies, participants, actors, and practices, which can strengthen the ties between science and the general public (31). It can empower individuals and communities, even the most marginalized ones, and support more inclusive scientific practices and outputs (32–34). It can help increase awareness and inspire action to address challenges facing our society in a collaborative way and guide decisions made at a local, national, and global level, encouraging more inclusive and responsive decision-making (35, 36). Citizen science can also help to address large data gaps and needs, crucial for both science and policy, including the monitoring of the SDGs, as mentioned above (29). Citizen science can also be referred to as crowdsourcing (37, 38), community-based monitoring (39), community-based participatory research (40), participatory action research (41), volunteered geographic information (42), and participatory sensing (43), among others, which reflects contextual, disciplinary and cultural differences in the field (44, 45). Citizen science approaches vary from contributory projects, where the role of the participants is to collect or analyze data, to co-created projects involving participants in most or all stages of research including study design (45, 46). Although citizen science is well established in environmental and ecological sciences as the field in which the term originated (47), it has also been gaining recognition in other disciplines and areas including health and well-being (48–52). For example, a recent study showed that citizen science approaches in the field of health have been used in chronic disease prevention to address various issues such as mental health, tobacco and alcohol control, nutrition and physical activity and green spaces (52). Other examples of health citizen science include self reporting on health conditions using wearables or mobile apps in cowdsourced health research (53, 54), mobilizing communities to support the monitoring of public service delivery in community-based monitoring projects (55), and sharing lived experiences in managing complex health conditions in patient-powered research networks (56). More examples of existing health citizen science projects are provided in the results section, as well as in the Supplementary material. The data gaps related to health and well-being are shown in Figures 1–3.

Some research has already been undertaken to examine the potential of citizen science for monitoring progress toward the SDGs (57–61). A systematic review undertaken by Fraisl et al. (62) at the indicator level focused on the potential of citizen science data for SDG monitoring and found that citizen science is already contributing to the monitoring of five SDG indicators and has the potential to contribute to an additional 76 indicators. Although the majority of contributions were situated in the environmental domain, SDG 3 on Health and Well-being, and SDG 6 on Clean Water and Sanitation were both identified as having a high potential for benefiting from citizen science data. The study also demonstrated that the WHO is the custodian agency that has the greatest potential in terms of benefiting from citizen science data for SDG monitoring (62).

Although some citizen science initiatives were identified that could address the data gaps and needs in the SDG GIF by Fraisl et al. (62), there is a need to gain a more comprehensive understanding of the potential of citizen science for contributing to those SDG indicators specifically related to health and well-being. This would not only help to better assess and act on the contributions of citizen science data to SDG monitoring and implementation, but it would also support the mobilization of resources, guiding action and creating multistakeholder partnerships, as highlighted in GPW13 and the Triple Billion Targets. It is important to note that well-being has not yet been properly addressed by WHO but work is underway to address that lacuna (63), which may lead to even further data demands. Additionally, identifying the potential contributions of citizen science data to measure and achieve the Triple Billion Targets requires a more in-depth investigation.

Hence, the aim of this paper is to update and extend the systematic review of Fraisl et al. (62), focusing only on health and well-being, to demonstrate the potential contributions and opportunities of citizen science data for achieving health and well-being related SDGs and Triple Billion Targets considering Covid-19 as a driver. The paper also aims to discuss the challenges raised by citizen science approaches. Finally, the paper provides recommendations that could be turned into actionable results and success stories that will set examples for NSOs and National Statistical Systems (NSS) worldwide to reduce their health and well-being related data gaps and mobilize timely action to achieve health and well-being for all.

## 2. Materials and methods

**Figure 4:**
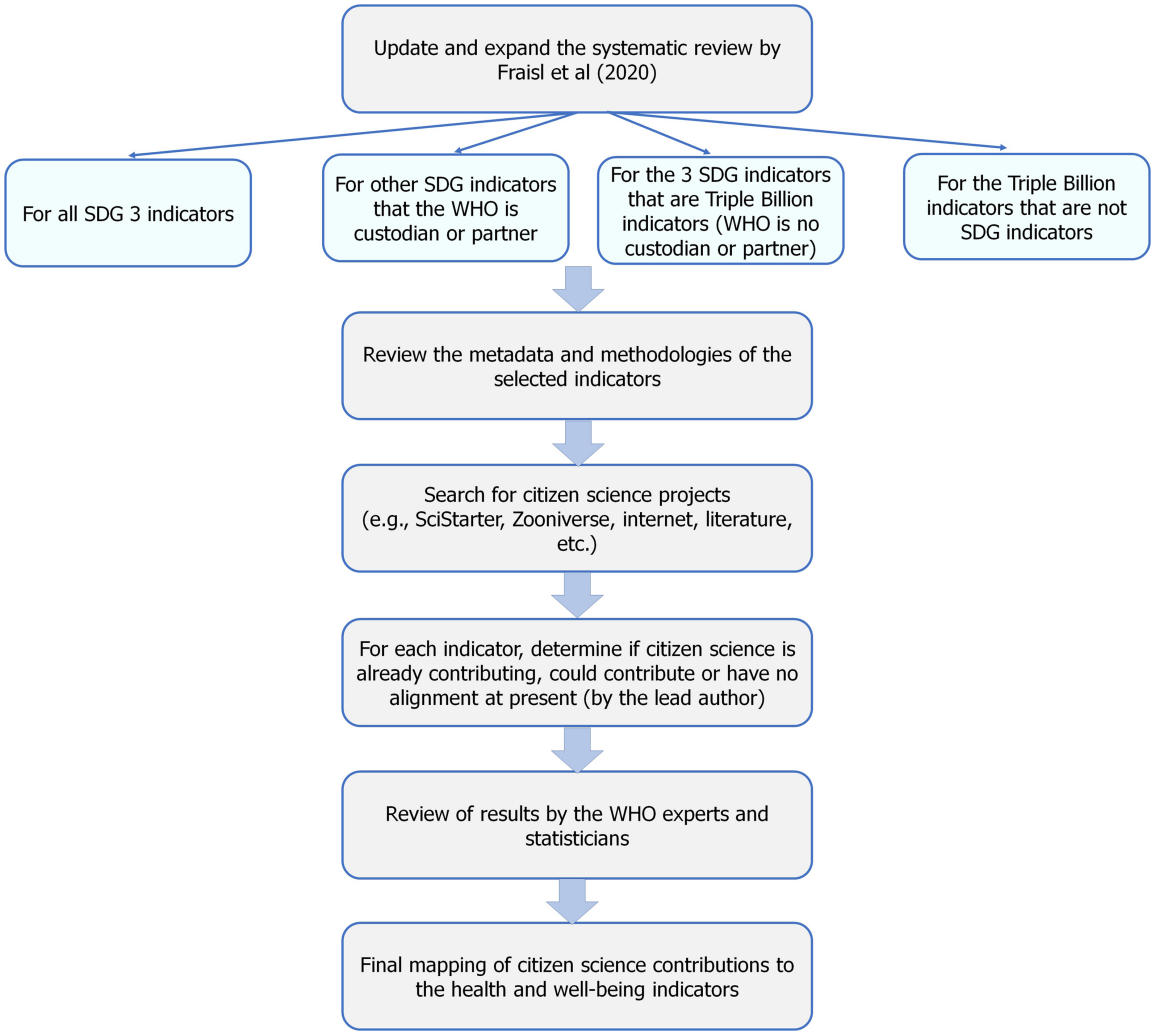
Overview of the methodology used in the study.

The methodology is composed of two parts. In the first part, we updated and expanded the systematic review that was undertaken by Fraisl et al. (62), focusing only on those SDG indicators related to health and well-being and added to it the additional WHO Triple Billion indicators that are not covered in the SDGs. The second part involved synthesizing results from internet and literature searches to gain a better understanding of where citizen science is currently contributing to health and well-being more generally. Figure 4 shows the various steps in the methodology.

The starting point for this health and well-being related indicator review with respect to relevant citizen science initiatives is Supplementary Table S1 in the Supplementary material of the study undertaken by Fraisl et al. (62). The first step was to filter the table for those SDG indicators that are related to health and well-being. This was done by first selecting all 28 indicators in SDG 3 Good Health and Well-being. Further indicators from other SDGs were then added in which WHO is listed as a custodian or partner agency. Following this step, three SDG indicators, i.e., 1.5.1 *Number of deaths, missing persons and directly affected persons attributed to disasters per 100,000 population*, 5.6.1 *Proportion of women aged 15–49 years who make their own informed decisions regarding sexual relations, contraceptive use and reproductive health care* and 16.2.1 *Proportion of children aged 1–17 years who experienced any physical punishment and/or psychological aggression by caregivers in the past month,* were added even though WHO is not listed as a custodian or partner agency, because they are part of the WHO Triple Billion Targets. Finally, seven additional indicators covered by the WHO Triple Billion Targets (which are not part of the SDG GIF) were added to this review, as all the other Triple Billion Targets are also SDG indicators at the same time and are already covered in the study because of this overlap. We then updated this table for changes to the tier classification using the latest version of the SDG indicators downloaded from the UN Statistics Division website corresponding to 30 November 2020. The final list of indicators covered in this analysis related to health and well-being contains 58 indicators in total. Table 1 provides a list of these indicators, their targets, goals and the latest tier classifications (updated on 30 November 2022 by the UNSD), which shows 51 SDG indicators, and seven additional Triple Billion indicators that are not covered in the SDG GIF.

**Table 1:** A list of health and well-being related indicators covered in this study as part of the SDG GIF in which the WHO is listed as a custodian or partner agency and the WHO’s Triple Billion Targets, as well as the brief results of the systematic review.

Target	Indicator	Current tier classification (2023)	Potential of citizen science
Goal 1. End poverty in all its forms everywhere			
1.4 By 2030, ensure that all men and women, in particular the poor and the vulnerable, have equal rights to economic resources, as well as access to basic services, ownership and control over land and other forms of property, inheritance, natural resources, appropriate new technology and financial services, including microfinance	1.4.1 Proportion of population living in households with access to basic services	Tier I	Could Contribute
1.5 By 2030, build the resilience of the poor and those in vulnerable situations and reduce their exposure and vulnerability to climate-related extreme events and other economic, social and environmental shocks and disasters	1.5.1 Number of deaths, missing persons and directly affected persons attributed to disasters per 100,000 population	Tier I	Could Contribute
1.a Ensure significant mobilization of resources from a variety of sources, including through enhanced development cooperation, in order to provide adequate and predictable means for developing countries, in particular least developed countries, to implement program and policies to end poverty in all its dimensions	1.a.2 Proportion of total government spending on essential services (education, health, and social protection)	Tier I/II depending on service	No alignment
Goal 2. End hunger, achieve food security and improved nutrition and promote sustainable agriculture			
2.2 By 2030, end all forms of malnutrition, including achieving, by 2025, the internationally agreed targets on stunting and wasting in children under 5 years of age, and address the nutritional needs of adolescent girls, pregnant and lactating women and older persons	2.2.1 Prevalence of stunting (height for age < −2 standard deviation from the median of the World Health Organization (WHO) Child Growth Standards) among children under 5 years of age	Tier I	No alignment
	2.2.2 Prevalence of malnutrition (weight for height > +2 or < −2 standard deviation from the median of the WHO Child Growth Standards) among children under 5 years of age, by type (wasting and overweight)	Tier I	Could Contribute
	2.2.3 Prevalence of anemia in women aged 15 to 49 years, by pregnancy status (percentage)	Tier I	Could Contribute
Goal 3. Ensure healthy lives and promote well-being for all at all ages			
3.1 By 2030, reduce the global maternal mortality ratio to less than 70 per 100,000 live births	3.1.1 Maternal mortality ratio	Tier I	Could Contribute
	3.1.2 Proportion of births attended by skilled health personnel	Tier I	Could Contribute
3.2 By 2030, end preventable deaths of newborns and children under 5 years of age, with all countries aiming to reduce neonatal mortality to at least as low as 12 per 1,000 live births and under-5 mortality to at least as low as 25 per 1,000 live births	3.2.1 Under-5 mortality rate	Tier I	Could Contribute
	3.2.2 Neonatal mortality rate	Tier I	Could Contribute
3.3 By 2030, end the epidemics of AIDS, tuberculosis, malaria and neglected tropical diseases and combat hepatitis, water-borne diseases and other communicable diseases	3.3.1 Number of new HIV infections per 1,000 uninfected population, by sex, age, and key populations	Tier I	Could Contribute
	3.3.2 Tuberculosis incidence per 100,000 population	Tier I	Could Contribute
	3.3.3 Malaria incidence per 1,000 population	Tier I	Could Contribute
	3.3.4 Hepatitis B incidence per 100,000 population	Tier I	Could Contribute
	3.3.5 Number of people requiring interventions against neglected tropical diseases	Tier I	Could Contribute
3.4 By 2030, reduce by one third premature mortality from non-communicable diseases through prevention and treatment and promote mental health and well-being	3.4.1 Mortality rate attributed to cardiovascular disease, cancer, diabetes or chronic respiratory disease	Tier I	Could Contribute
	3.4.2 Suicide mortality rate	Tier I	Could Contribute
3.5 Strengthen the prevention and treatment of substance abuse, including narcotic drug abuse and harmful use of alcohol	3.5.1 Coverage of treatment interventions (pharmacological, psychosocial and rehabilitation and aftercare services) for substance use disorders	Tier II	Could Contribute
	3.5.2 Alcohol *per capita* consumption (aged 15 years and older) within a calendar year in liters of pure alcohol	Tier I	Could Contribute
3.6 By 2020, halve the number of global deaths and injuries from road traffic accidents	3.6.1 Death rate due to road traffic injuries	Tier I	Could Contribute
3.7 By 2030, ensure universal access to sexual and reproductive health-care services, including for family planning, information and education, and the integration of reproductive health into national strategies and program	3.7.1 Proportion of women of reproductive age (aged 15–49 years) who have their need for family planning satisfied with modern methods	Tier I	Could Contribute
	3.7.2 Adolescent birth rate (aged 10–14 years; aged 15–19 years) per 1,000 women in that age group	Tier I	Could Contribute
3.8 Achieve universal health coverage, including financial risk protection, access to quality essential health-care services and access to safe, effective, quality and affordable essential medicines and vaccines for all	3.8.1 Coverage of essential health services	Tier I	Could Contribute
	3.8.2 Proportion of population with large household expenditures on health as a share of total household expenditure or income	Tier I	No alignment
3.9 By 2030, substantially reduce the number of deaths and illnesses from hazardous chemicals and air, water and soil pollution and contamination	3.9.1 Mortality rate attributed to household and ambient air pollution	Tier I	Could Contribute
	3.9.2 Mortality rate attributed to unsafe water, unsafe sanitation and lack of hygiene (exposure to unsafe Water, Sanitation and Hygiene for All (WASH) services)	Tier I	Could Contribute
	3.9.3 Mortality rate attributed to unintentional poisoning	Tier I	Could Contribute
3.a Strengthen the implementation of the World Health Organization Framework Convention on Tobacco Control in all countries, as appropriate	3.a.1 Age-standardized prevalence of current tobacco use among persons aged 15 years and older	Tier I	Could Contribute
3.b Support the research and development of vaccines and medicines for the communicable and non-communicable diseases that primarily affect developing countries, provide access to affordable essential medicines and vaccines, in accordance with the Doha Declaration on the TRIPS Agreement and Public Health, which affirms the right of developing countries to use to the full the provisions in the Agreement on Trade-Related Aspects of Intellectual Property Rights regarding flexibilities to protect public health, and, in particular, provide access to medicines for all	3.b.1 Proportion of the target population covered by all vaccines included in their national program	Tier I	Could Contribute
	3.b.2 Total net official development assistance to medical research and basic health sectors	Tier I	No alignment
	3.b.3 Proportion of health facilities that have a core set of relevant essential medicines available and affordable on a sustainable basis	Tier II	Could Contribute
3.c Substantially increase health financing and the recruitment, development, training and retention of the health workforce in developing countries, especially in least developed countries and small island developing States	3.c.1 Health worker density and distribution	Tier I	No alignment
3.d Strengthen the capacity of all countries, in particular developing countries, for early warning, risk reduction and management of national and global health risks	3.d.1 International Health Regulations (IHR) capacity and health emergency preparedness	Tier I	No alignment
	3.d.2 Percentage of bloodstream infections due to selected antimicrobial-resistant organisms	Tier II	Could Contribute
Goal 4. Ensure inclusive and equitable quality education and promote lifelong learning opportunities for all			
4.2 By 2030, ensure that all girls and boys have access to quality early childhood development, care and pre-primary education so that they are ready for primary education	4.2.1 Proportion of children aged 24–59 months who are developmentally on track in health, learning and psychosocial well-being, by sex	Tier II	Could Contribute
Goal 5. Achieve gender equality and empower all women and girls			
5.2 Eliminate all forms of violence against all women and girls in the public and private spheres, including trafficking and sexual and other types of exploitation	5.2.1 Proportion of ever-partnered women and girls aged 15 years and older subjected to physical, sexual or psychological violence by a current or former intimate partner in the previous 12 months, by form of violence and by age	Tier I	Already Contributing & Could Contribute
	5.2.2 Proportion of women and girls aged 15 years and older subjected to sexual violence by persons other than an intimate partner in the previous 12 months, by age and place of occurrence	Tier II	Already Contributing & Could Contribute
5.3 Eliminate all harmful practices, such as child, early and forced marriage and female genital mutilation	5.3.1 Proportion of women aged 20–24 years who were married or in a union before age 15 and before age 18	Tier I	Could Contribute
	5.3.2 Proportion of girls and women aged 15–49 years who have undergone female genital mutilation/cutting, by age	Tier I	Could Contribute
5.6 Ensure universal access to sexual and reproductive health and reproductive rights as agreed in accordance with the Program of Action of the International Conference on Population and Development and the Beijing Platform for Action and the outcome documents of their review conferences	5.6.1 Proportion of women aged 15–49 years who make their own informed decisions regarding sexual relations, contraceptive use and reproductive health care	Tier II	Already Contributing & Could Contribute
	5.6.2 Number of countries with laws and regulations that guarantee full and equal access to women and men aged 15 years and older to sexual and reproductive health care, information and education	Tier I	No alignment
Goal 6. Ensure availability and sustainable management of water and sanitation for all			
6.1 By 2030, achieve universal and equitable access to safe and affordable drinking water for all	6.1.1 Proportion of population using safely managed drinking water services	Tier I	Could Contribute
6.2 By 2030, achieve access to adequate and equitable sanitation and hygiene for all and end open defecation, paying special attention to the needs of women and girls and those in vulnerable situations	6.2.1 Proportion of population using (a) safely managed sanitation services and (b) a hand-washing facility with soap and water	Tier I (a)/Tier II (b)	Could Contribute
6.3 By 2030, improve water quality by reducing pollution, eliminating dumping and minimizing release of hazardous chemicals and materials, halving the proportion of untreated wastewater and substantially increasing recycling and safe reuse globally	6.3.1 Proportion of domestic and industrial wastewater flows safely treated	Tier II	Could Contribute
6.a By 2030, expand international cooperation and capacity-building support to developing countries in water-and sanitation-related activities and programs, including water harvesting, desalination, water efficiency, wastewater treatment, recycling and reuse technologies	6.a.1 Amount of water-and sanitation-related official development assistance that is part of a government-coordinated spending plan	Tier I	No alignment
6.b Support and strengthen the participation of local communities in improving water and sanitation management	6.b.1 Proportion of local administrative units with established and operational policies and procedures for participation of local communities in water and sanitation management	Tier I	No alignment
Goal 7. Ensure access to affordable, reliable, sustainable and modern energy for all			
7.1 By 2030, ensure universal access to affordable, reliable and modern energy services	7.1.2 Proportion of population with primary reliance on clean fuels and technology	Tier I	Could Contribute
Goal 11. Make cities and human settlements inclusive, safe, resilient and sustainable			
11.6 By 2030, reduce the adverse *per capita* environmental impact of cities, including by paying special attention to air quality and municipal and other waste management	11.6.2 Annual mean levels of fine particulate matter (e.g., PM2.5 and PM10) in cities (population weighted)	Tier I	Could Contribute
Goal 16. Promote peaceful and inclusive societies for sustainable development, provide access to justice for all and build effective, accountable and inclusive institutions at all levels			
16.1 Significantly reduce all forms of violence and related death rates everywhere	16.1.1 Number of victims of intentional homicide per 100,000 population, by sex and age	Tier I	Could Contribute
	16.1.3 Proportion of population subjected to (a) physical violence, (b) psychological violence and (c) sexual violence in the previous 12 months	Tier II	Already Contributing & Could Contribute
16.2 End abuse, exploitation, trafficking and all forms of violence against and torture of children	16.2.1 Proportion of children aged 1–17 years who experienced any physical punishment and/or psychological aggression by caregivers in the past month	Tier II	Could Contribute
Triple Billion Target: Health emergencies	Vaccine coverage for epidemic prone diseases	N/A	Could Contribute
Triple Billion Target: Health emergencies	Proportion of vulnerable people in fragile settings provided with essential health services (%)	N/A	Could Contribute
Triple Billion Target: Healthier populations	WHA66.10: Prevalence of raised blood pressure in adults aged ≥18	N/A	Could Contribute
Triple Billion Target: Healthier populations	WHA66.10: Best practice policy implemented for industrially produced trans fatty acids (TFA) (Y/N)	N/A	No alignment
Triple Billion Target: Healthier populations	WHA66.10: Prevalence of obesity among children and adolescents (aged 5–19) (%) Prevalence of obesity among adults aged ≥18	N/A	Could Contribute
Triple Billion Target: Healthier populations	N WHA68.3: Number of cases of poliomyelitis caused by wild poliovirus	N/A	Could Contribute
Triple Billion Target: Healthier populations	WHA68.7: Patterns of antibiotic consumption at national level	N/A	Could Contribute

In the next phase, we consulted the methodologies of these indicators by reviewing the metadata documents of health and well-being related SDG indicators for any update in the methodology that may have occurred since the systematic review of Fraisl et al. (62) was undertaken, i.e., if they all had agreed methodologies at the time of the review. Then, we examined the methodologies of seven non-SDG indicators that are Triple Billion Target indicators described in the GPW13.

Following this step, we systematically searched for additional citizen science initiatives to those identified by Fraisl et al. (62). Our search included the following:

1. We queried two databases of citizen science projects: Scistarter^1^ and Zooniverse^2^ using health and well-being, and indicator specific keywords, such as air quality, water quality, etc.;
2. We did an internet search using health and well-being and indicator specific keywords and combinations of ‘citizen science’, ‘community-based monitoring’, ‘crowdsourcing’, etc. and found further literature through reference lists in papers and reports. For example, for indicator “3.3.3 Malaria incidence per 1,000 population,” the keyword search included “citizen science” AND “malaria”; “citizen science” AND “mosquito”; “community-based monitoring” AND “malaria”; “community-based monitoring” AND “mosquito”; “crowdsourcing” AND “malaria” and others. The internet search was necessary because many of these citizen science initiatives, especially local scale and community level projects, such as community-based monitoring activities, do not appear in the peer-reviewed literature.

In contrast to Fraisl et al. (62), where the objective was to find at least one example of a citizen science project contributing to the SDGs or with the potential to contribute, here we attempted to find more projects to have a better overview of the landscape of relevant current and past initiatives. In terms of the exclusion criteria, one example is that similar to Fraisl et al. (62), we did not include projects where participants were paid salaries such as some Community Health Workers (CHWs), but we did include those where participants may receive small incentives such as uniforms and bikes to be able to commute between villages (64). One challenge regarding the inclusion or exclusion of projects in this study was related to the definitions and terminologies related to both “citizen science” and “health and well-being.” As highlighted in the introduction section, setting concrete boundaries for the field of citizen science is difficult due to diverse terms, definitions, methodologies and approaches involved, which also makes it difficult to decide which activities fall under the umbrella of citizen science. To address this challenge, we used the European Citizen Science Association (ECSA) Characteristics of Citizen Science, which aim to assist in the interpretation of citizen science activities regarding what constitutes citizen science (45, 65, 66). Similarly, defining health and well-being is also not very straightforward. In the context of this review, our inclusion and exclusion criteria included the topics, targets and indicators covered by SDG 3 “Good Health and Well-being” as well as other public, global and environmental health and well-being issues that are subject to other SDGs and Triple Billion Targets for which the WHO is custodian or partner agency as described in the introduction section. Finally, as an inclusion criteria, we selected initiatives that are in English, as the working language of the authors is English, which is a limitation addressed in the discussion section.

We then reviewed each health and well-being indicator to explore whether the newly identified citizen science projects (i) are currently contributing data to an indicator, which refers to the cases where the data from a citizen science project are or were used for monitoring or reporting of a specific indicator at a national or global level; or (ii) could contribute to them in the future, which is used to describe projects, where data from a citizen science initiative could be used to monitor an indicator, but are currently not used and the project methodology may need to be revised before the data can be used for monitoring and reporting purposes; or (iii) no alignment was identified with any existing or ongoing citizen science initiative and the indicator. It is important to note that this review is based on existing citizen science initiatives, which means that for an indicator that is mapped as “no alignment” in this study, a new citizen science project may be available in the future and our results may need to be updated. The definitions and criteria for each of these three categories are identified by Fraisl et al. (62). Additionally, both the SDGs and the Triple Billion Targets are interlinked, which means that a project can potentially contribute to several indicators, as shown in the Supplementary material. For example, the “Snakebite Information and Data Platform” project “could contribute” to the monitoring of several indicators, such as 1.4.1, 3.3.5, and the Triple Billion indicator *Health emergencies: Proportion of vulnerable people in fragile settings provided with essential health services* (see the Supplementary material for details).

If a project was found that “could contribute,” we then examined (ii) whether this contribution is direct or supplementary. Direct contributions are those in which citizen science data are already contributing or could contribute to the calculation of the official SDG indicator, e.g., as a primary data source, while supplementary contributions are those in which the data from citizen science could provide useful contextual information for better understanding an indicator, as implemented by Fraisl et al. (62). For example, for the indicator 5.2.1 *Proportion of ever-partnered women and girls aged 15 years and older subjected to physical, sexual or psychological violence by a current or former intimate partner in the previous 12 months, by form of violence and by age*, we mapped the “Let us Talk” project (67–69), where users anonymously record information related to gender based violence, by the Ministry of Gender, Children, and Social Protection in Ghana and the Ghana Statistical Service, as a *direct contribution*. The data collected through this initiative can be used directly for the monitoring of 5.2.1 due to the direct relevance of the project to the indicator. Additionally, the GSS has highlighted the project for 5.2.1 in their latest Voluntary National Review (VNR), which is a UN process allowing countries to assess and present their national level progress in implementing the SDGs (69). The indicators that had no foreseen contribution at present were revisited to confirm this status. Once this review was completed, we then summarized the results to understand the present status of health and well-being citizen science initiatives in relation to their contributions to the SDGs and the Triple Billion Targets.

## 3. Results

**Figure 5:**
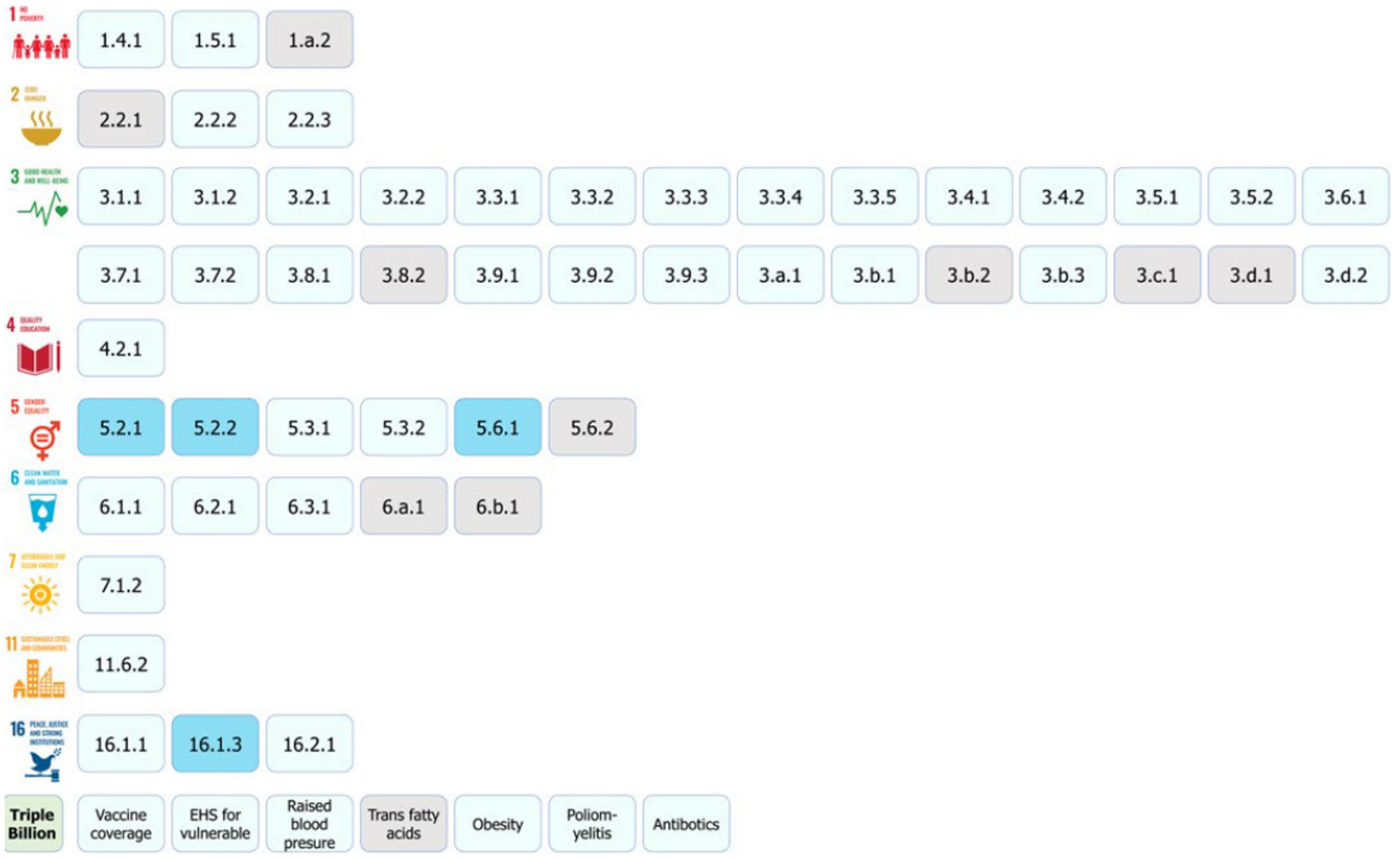
The health and well-being related SDG indicators and Triple Billion Targets covered in this study. Indicators where citizen science projects are “already contributing” are in dark blue, “could contribute” are in light blue or where there is “no alignment” are in grey. The values within each box are the indicator numbers or titles.

**Figure 6:**
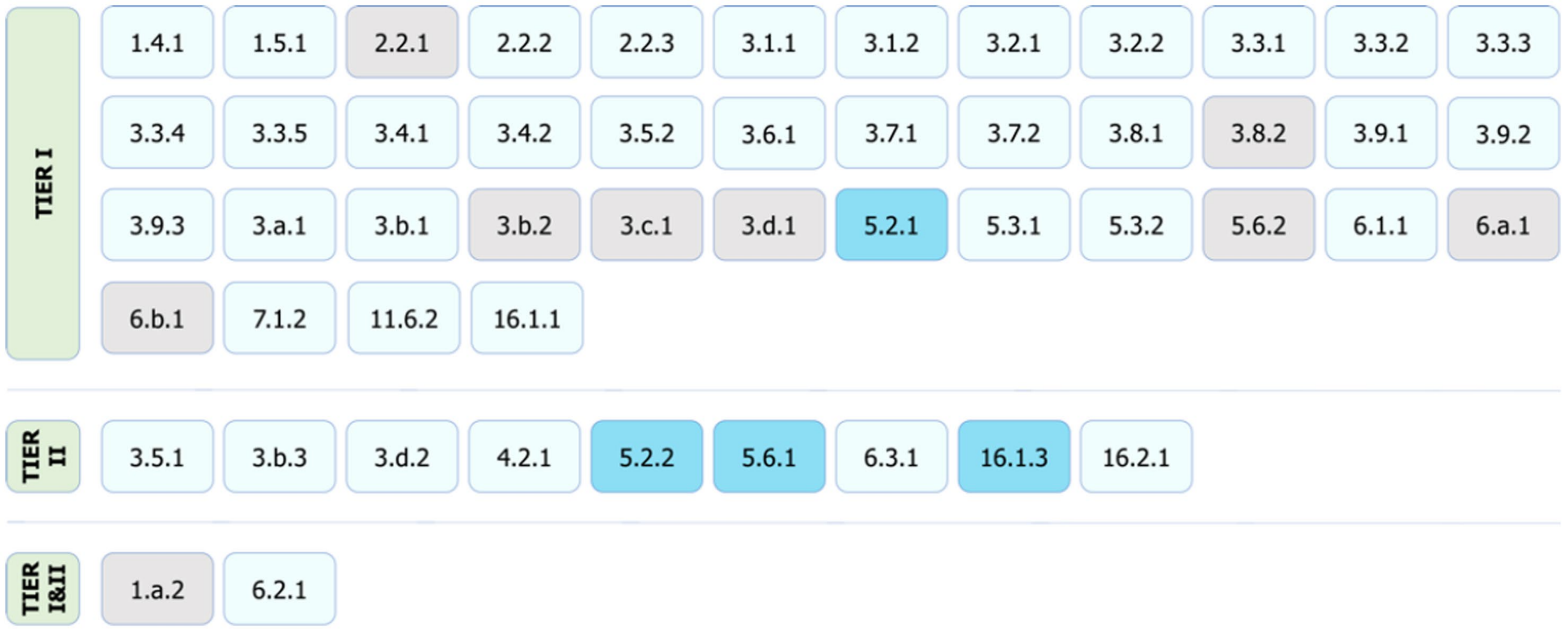
Citizen science contributions to SDG monitoring by tier classification. The light (could contribute) and dark (already contributing) blue shading denote direct or supplementary contributions, and the grey shading shows no alignment. The values within each box are the SDG indicator numbers.

**Figure 7:**
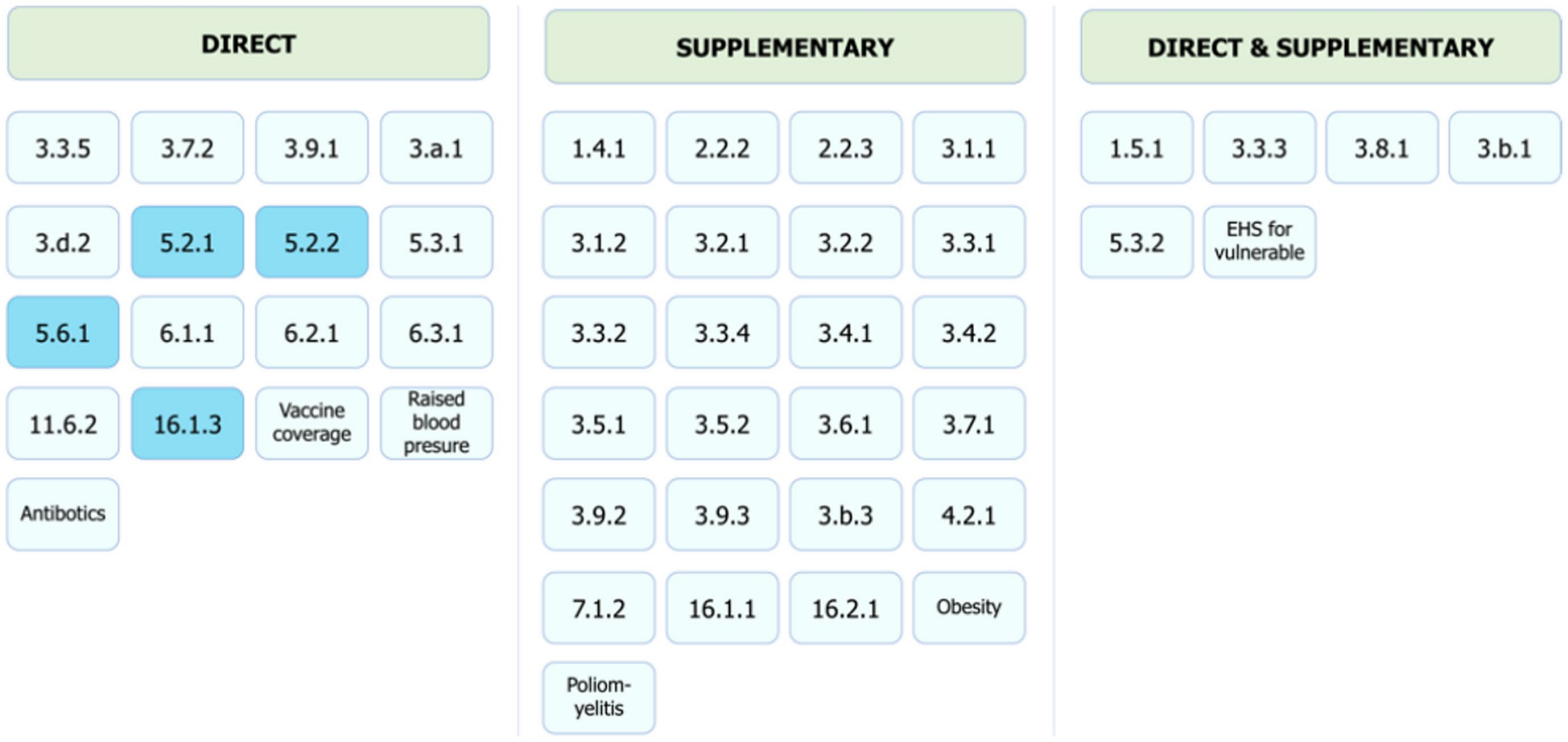
Direct, supplementary and both direct and supplementary contributions of citizen science to SDG and Triple Billion Targets monitoring. Indicators where citizen science projects are “already contributing” are in dark blue and “could contribute” in light blue. The values within each box are indicator numbers or titles.

Our results show that out of 58 health and well-being related indicators, including the SDGs and the Triple Billion Targets, citizen science has the potential to contribute to the monitoring of 48 indicators in a direct or supplementary way or support the achievement of their targets mostly at a community/local level. Hence, our findings indicate that citizen science data can contribute to the monitoring of 83% of health and well-being related indicators. Table 1 shows a summary of contributions as per indicator. Figures 5–7 summarize the results. The full review and the detailed results, which present the potential of citizen science initiatives by health and well-being related indicator, can be found in Supplementary Table S1 of the Supplementary material.

Based on the current SDG tier classification (30 November 2022), 40 out of the 51 health and well-being related SDG indicators covered in this study are Tier I, 9 are Tier II, and 2 have both Tier I and Tier II components. Our results show that, although most of the SDG indicators with potential for benefiting from citizen science are Tier I (31 out of 40 Tier I health and well-being related SDG indicators) (Figure 6), meaning that the data are mostly available as official statistics, there are still big gaps in data availability and timeliness for those Tier I indicators, as well. For example, for around one third of countries, the GPW13 framework highlights that there is no recent primary or underlying data for more than half of the SDG health and well-being related indicators (9). This shows that, regardless of the tier classification, large data gaps exist in health and well-being related SDGs, some of which could be addressed through citizen science initiatives designed, improved and/or upscaled based on relevant considerations such as disaggregating data by sex, age, location, economic status, and education, among others.

In terms of direct or supplementary contributions (Figure 7), our findings indicate that of the 48 indicators that could benefit from citizen science approaches, these contributions can be direct for 17 indicators, supplementary for 25 indicators and both direct and supplementary for 6 indicators. Supplementary Table S1 of Supplementary material shows the indicators by the latest tier classification and their potential for benefiting from citizen science data in terms of direct and supplementary contributions.

In the specific case of SDG 3, our results also show that out of 28 indicators, citizen science data can contribute to the monitoring of 24 indicators (Table 1; Figure 5), which equates to 85% of SDG 3 indicators. Some of these indicators were identified previously in the systematic review by Fraisl et al. (62); examples include 3.1.1 *Maternal mortality ratio*, 3.3.3 *Malaria incidence per 1,000 population* and 3.9.1 *Mortality rate attributed to household and ambient air pollution*, among others. However, due to the more comprehensive and focused approach undertaken here in which only health and well-being related indicators were considered, other indicators have now been identified, such as 3.4.1 *Mortality rate attributed to cardiovascular disease, cancer, diabetes or chronic respiratory disease*, 3.4.2 *Suicide mortality rate* and 3.9.2 *Mortality rate attributed to unsafe water, unsafe sanitation and lack of hygiene (exposure to unsafe Water, Sanitation and Hygiene for All (WASH) services)*.

Based on the results found here, we now provide some examples in the following section for some randomly selected indicators to demonstrate which citizen science initiatives could contribute to which health and well-being related indicators and targets.

### 3.1. Indicator 2.2.2 Prevalence of malnutrition (weight for height > +2 or < −2 standard deviation from the median of the WHO Child Growth Standards) among children under 5 years of age, by type (wasting and overweight)

Indicator 2.2.2 is under SDG Target 2.2 *By 2030, end all forms of malnutrition, including achieving, by 2025, the internationally agreed targets on stunting and wasting in children under 5 years of age, and address the nutritional needs of adolescent girls, pregnant and lactating women and older persons.* This is also a Triple Billion indicator under the Healthier Populations target: *1 billion more people enjoying better health and well-being*.

This indicator has 2 subindicators: (i) 2.2.2a *Prevalence of overweight (weight for height > +2 standard deviation from the median of the World Health Organization (WHO) Child Growth Standards) among children under 5 years of age* and (ii) 2.2.2b *Prevalence of wasting (weight for height <−2 standard deviation from the median of the World Health Organization (WHO) Child Growth Standards) among children under 5 years of age”.*

For 2.2.2a, BigO (70–72) is an example that demonstrates how citizen science approaches could provide supplementary contributions for monitoring Target 2.2, even though its target group is school aged children. In BigO, the objective is to collect and analyze anonymous data on children’s behavioral patterns and their living environment using the myBigOapp. With advanced analytics, BigO gathers evidence on which local factors contribute to childhood obesity in Europe, and how they affect it. These data are anonymized and used to create statistical models to understand how behavior and the environment influence the prevalence of childhood obesity including its underlying factors. If BigO could be adapted to target children under 5 years of age through their families, it could provide direct contribution to the monitoring of this indicator (71).

Another initiative is a community-based growth monitoring model in a mountainous rural village of South Africa, where access to health facilities are limited. The objective was to assess the feasibility of a community-based growth monitoring model in alleviating the issues in health and nutrition surveillance of preschool-aged children. The results showed that community participation and mobilization can increase preschool child growth monitoring coverage and help to achieve improved health and nutrition surveillance. The results are important in terms of demonstrating the potential of such initiatives in understanding malnutrition among preschool aged children at a community level (73).

EPODE (74, 75), “Ensemble Prevenons l’Obesité Des Enfants,” meaning “Together let us prevent childhood obesity” is another program that could support this indicator and the relevant target. It was implemented in two communities in northern France. The aim was to empower those communities to combat and prevent childhood obesity. As part of the program, a local steering committee was mobilized in order to place the community at the heart of the EPODE system. Community-based monitoring or citizen science initiatives like EPODE can help mobilize local actions to address the global issue of child obesity (2.2.2.a) or malnutrition (2.2.2.b), and support both monitoring of the relevant target and outcomes of program implementation (74).

### 3.2. Indicator 3.3.5 Number of people requiring interventions against neglected tropical diseases

Indicator 3.3.5 is under SDG Target 3.3 *By 2030, end the epidemics of AIDS, tuberculosis, malaria and neglected tropical diseases and combat hepatitis, water-borne diseases and other communicable diseases*. It is also a Triple Billion indicator under the Universal Health Coverage target: *one billion more people benefiting from universal health coverage*.

The WHO initiative “Snakebite Information and Data Platform” (25) can provide useful information to support this indicator, as well as the achievement of the relevant target as the WHO added snakebite envenomation to Category A of the Neglected Tropical Diseases in 2017 (76). The platform aims to contribute to achieving the global target to “halve the number of deaths and disability due to snakebite envenoming by 2030”. The platform also aims to improve surveillance and contribute to related epidemiological documentation and data, which countries can use to compile statistics for SDG monitoring and beyond. This approach allows for better integration of data to improve the mapping and distribution of antivenoms, as well as sharing of resources and coordination of prevention activities. The platform allows the public to participate and contribute by sharing photos of what they think are venomous snakes along with their location data. It also shows where the antivenom treatments are needed, which can allow prompt citizen access to treatment. Hence, the project provides an example of how citizen science data along with other information can enable access to targeted healthcare (77).

### 3.3. Indicator 3.6.1 Death rate due to road traffic injuries

Indicator 3.6.1 is under SDG Target 3.6 *By 2020, halve the number of global deaths and injuries from road traffic accidents*. This indicator is also a Triple Billion indicator under the Healthier Populations target: *one billion more people enjoying better health and well-being*. The road injury estimates are based on death registration data and reported road traffic deaths from official road traffic surveillance systems gathered in a WHO survey of Member States. Road injury deaths were projected forward to 2019 using recent trends in death registration data if available, or the trend for recent years to 2019 from the Global Burden of Disease 2019 (GBD2019) (9, 78).

“Road traffic injury prevention training manual” by WHO and the Indian Institute of Technology highlights that gathering data on road traffic injuries through conducting community-based surveys is one approach for understanding the extent of the problem. This is because some injured patients do not reach hospitals for a variety of reasons, in which case they will not be registered in hospital-based injury surveillance systems (79).

For example, in a study that developed and implemented an Integrated Road Traffic Injuries (RTI) Surveillance in India, an electronic-based comprehensive and integrated RTI surveillance system was established. The system included one trauma center, one private hospital and a community with a population of 10 K in urban areas. In rural areas, a district hospital, a private nursing home and two sub-center areas of different primary health centers at each site were part of the project. Active surveillance was placed in communities to track missing cases. The results showed that such a model including both passive and active surveillance, which includes community members, is helpful to collect data on the maximum number of road traffic injuries (80).

In another study that aimed to develop a framework for injury surveillance in Canadian Aboriginal communities, an injury surveillance framework that is culturally relevant and acceptable by the community was created. The results showed that injury surveillance systems are usually under the control of the authorities instead of the communities themselves. (81).

Citizen science projects related to road safety, even though not related to monitoring death rate, can provide additional supplementary information to contextualize the road injuries and safety issues. For example, the Bike Barometer project from Flanders, Belgium, where 1,256 adolescents from 31 schools recorded 5,657 km of roads, of which 3,750 km were evaluated for cycling friendliness and safety, can provide insights into local safety conditions for Flanders and for specific school neighborhoods and is highly relevant for local decision-making (82). In a similar project, “SimRa” short for “Safety in Bicycle Traffic” from Berlin, using a smartphone app, data about near crashes and main routes of bicycle traffic are collected. These data can then be used to develop recommendations for transportation policy and urban development measures (83).

Even though the indicator aims to measure death rate, the above-mentioned and other similar projects cannot only provide useful data on potential injuries or deaths, but also help mobilize action toward the achievement of the target.

### 3.4. Indicator 5.2.1 Proportion of ever-partnered women and girls aged 15 years and older subjected to physical, sexual, or psychological violence by a current or former intimate partner in the previous 12 months, by form of violence and by age

Indicator 5.2.1 is under SDG Target 5.2 *Eliminate all forms of violence against all women and girls in the public and private spheres, including trafficking and sexual and other types of exploitation*. 5.2.1 is also a Triple Billion indicator under the Health Populations Target: *1 billion more people to enjoy better health and well-being*.

The Ghana Statistical Service (GSS) has used citizen science approaches to collect data related to this indicator, as well as the indicator 5.2.2 *Proportion of women and girls aged 15 years and older subjected to sexual violence by persons other than an intimate partner in the previous 12 months, by age and place of occurrence,* among other relevant indicators. As part of the project, a pilot activity was conducted in three districts across all 3 ecological zones of Ghana (Ho, Techiman, and Central Gonja) and the project is planned to be rolled-out nationwide. In the project, through a design-thinking process, an app and an Interactive Voice Response (IVR) service, called Let us Talk, was developed, where users anonymously recorded information related to gender based violence. Results were compared to the data collected through traditional means and showed that through such approaches, more data can be collected, as women may not officially report on the violence they face. One of the advantages of citizen science approaches identified by the GSS through the project was that people are willing to report on behalf of others’ experiences of gender based violence, which is not possible through official surveys and official domestic violence reports in Ghana (67, 69).

### 3.5. Indicator 6.2.1 Proportion of population using (a) safely managed sanitation services and (b) a hand-washing facility with soap and water

Indicator 6.2.1 is under SDG Target 6.2 *By 2030, achieve access to adequate and equitable sanitation and hygiene for all and end open defecation, paying special attention to the needs of women and girls and those in vulnerable situations*. This indicator is also a Triple Billion indicator under the Healthier Populations target: *1 billion more people enjoying better health and well-being*.

The Sanitary Inspections (SI) in Malawi project can contribute to this indicator and target. As part of the project, a mixed methods approach of quantitative on-site and remote SI data collection via photographs, together with qualitative data collected by citizen scientists and a panel of experts were used. The results showed that the potential exists for citizens to conduct SI, with remote expert verification of the results using photographic images (84).

In another project, a citizen-science, climate justice planning process in the Mukuru informal settlement of Nairobi, Kenya was implemented. As part of the project, a data-gathering process with citizens was co-created and the project generated evidence to inform an integrated, climate justice strategy called the Mukuru Special Planning Area, Integrated Development Plan. The citizen science processes showed that <1% of residents had access to a private in-home toilet, and 37% lacked regular access to safe and affordable drinking water. As part of the project, thousands of residents were involved and they co-designed climate change adaptation strategies, such as flood mitigation, formalizing roads and pathways with drainage, and a water and sanitation infrastructure plan for all. Citizen scientists then used these data and moved this evidence into action to protect human health and drafted a climate justice strategy (85).

## 4. Discussion

Our research represents the first study covering health and well-being related SDG indicators and the WHO’s Triple Billion Targets that reviews the potential of citizen science to contribute to their monitoring. Previous studies have either covered the topic of an individual health and well-being related indicator, such as the potential of citizen science for monitoring air quality (86, 87), or the entire SDG GIF (62). However, these were not comprehensive or detailed enough to provide an in-depth analysis of the potential of citizen science for addressing health and well-being related indicators and targets. Additionally, they did not take the data gaps and monitoring challenges posed by the COVID-19 pandemic into account. COVID 19 was indeed considered as a driver of this study, not only in terms of substantially disrupting the achievement of the health and well-being targets and indicators, but also for the additional monitoring challenges it caused. Citizen science can help to address these challenges. For example, the Zoe Health Study, previously the Zoe Covid Study, is a project that aims to track the symptoms and spread of COVID-19, among other relevant objectives, through citizen science approaches. With over 5 million contributors globally, the project is the world’s largest ongoing study of COVID-19 (88, 89). In a different study called Outbreaks Near Me, epidemiologists and software developers at Harvard and Boston Children’s Hospital, along with a group of volunteers from across the technology industry, use citizen science data to create maps that help citizens and public health agencies identify existing and potential hotspots for COVID-19, and the annual influenza (90). The Zoe Health Study and Outbreaks Near Me, among many other citizen science projects, can help track the spread of COVID-19, identify who is most at risk and inform decisions that can save lives.

Our results show an increased potential for the contribution of citizen science data to health and well-being related indicators when compared to Fraisl et al. (62). For example, Fraisl et al. (62) identified that 15 out of 27 SDG 3 indicators (or 56%) *could benefit* from citizen science data. Here we identified an additional eight indicators that could benefit from citizen science, which equates to 24 out of 28 SDG 3 indicators, where one new indicator was added to SDG 3 after the study by Fraisl et al. (62). This increase is a result of the more focused research undertaken in this study covering only health and well-being related indicators.

Our results also highlight that even though citizen science is more dominant in the field of environmental monitoring, and thus has higher potential to contribute to the environmental SDG indicators, the health and well-being domain appears to be another area that can largely benefit from citizen science approaches, both in terms of monitoring and action (62, 91). We also identified that in the health domain, potential contributions are mostly from community-based initiatives that focus on communities in a particular country or district to address a specific issue faced by these communities. For example, the Global Challenges Research Fund (GCRF) Action Against Stunting Hub aimed to decrease child stunting by up to 10% in over 50 communities in India, Indonesia and Senegal (92). Such initiatives require a more targeted approach, where the project teams work with specific communities that are or can be affected by an issue, rather than publishing an open call to invite everyone to contribute data, knowledge, and observations, such as monitoring biodiversity or classifying satellite images to identify deforestation, which are mostly considered as contributory or crowdsourcing projects (44, 45).

Our findings also suggest that citizen science offers various opportunities for health and well-being monitoring processes. These include filling data gaps in existing data sets, providing useful complementary data outside traditional settings, delivering a more comprehensive overview of individual and public health in a timelier and cost-effective way, helping to triangulate existing data collected through traditional or non-traditional data sources, and supporting behavioral change by promoting healthy lifestyle choices such as walking or biking over driving, or raising awareness of societal issues such as child marriage and sexual harassment, among others. However, using citizen science data, as with any other traditional or new sources of data, has some challenges, as well. Some of these challenges are related to the accuracy, access, quality and integration with traditional sources of data, the standards for data collection, and recruiting and retaining participants, among others (29, 59). Nevertheless, many citizen science initiatives have addressed these issues in their data management lifecycles, e.g., by having quality assurance and quality control processes in place, including the use of multiple methods to ensure high quality data, and by having dedicated staff members for communication and outreach activities with participants (49). Demonstrating these measures and communicating them openly are key to overcoming some of the barriers to the use of data from citizen science for official statistics.

An important issue related to the use of citizen science for monitoring health and well-being or other global targets more generically is that citizen science initiatives usually follow different methodologies even when they aim to address data gaps on similar topics, such as the Mosquito Alert projects that aim to monitor mosquitoes of epidemiological interest. Aligning methodologies among citizen science initiatives can help to make data comparable, and thus their uptake for official monitoring. Initiatives such as the Citizen Science Global Partnership, a network of networks that aims to leverage citizen science to achieve sustainable development, can play a key role in addressing this issue by bringing together different citizen science projects conducting research on similar issues and help them align their methodologies with global methodologies (93). Another initiative that can help with the uptake of citizen science data for monitoring and achieving the health and well-being related goals and targets is the United Nations Development Program (UNDP) supported Accelerator Labs. These are a global network that aims to facilitate experiences gained in sustainable development, focusing on pressing and underachieved SDGs in the Global South; citizen science is one of the key approaches implemented as part of the initiative (94). The United Nations Statistics Division’s (UNSD) Collaborative on Citizen Data, which aims to establish a conceptual framework on citizen science and citizen-generated data for monitoring and achieving the SDGs, is another platform that can help with integrating citizen science approaches into the monitoring of health and well-being and other sustainable development indicators (95).

We are aware that data from citizen science are not perfect, but no data source is. The importance is to understand issues, challenges and biases related to their use and try to minimize them. Moreover, we are not suggesting that citizen science data should replace official monitoring activities. Rather, they can complement traditional data collection processes by providing additional data to better understand complex issues along with their context, reach out to those that may not be counted by official statistics, thus making monitoring more inclusive. Citizen science can also help to triangulate the data and provide additional verification for official statistics.

Citizen science is one of a set of ‘new data’ sources, and as such, it has an importance beyond the data themselves. The relative space occupied by official statistics coming from NSOs and from the UN and other international organizations in the information value chain is changing. Data, once seen primarily as an input for statistics, is now understood to be part of a host of rapidly expanding ecosystems and economies. This change in the role and conceptualization of data has been paralleled by an awakening of governments to the importance of data and of official statistics, contributing to a steady politicization of the field (96). Thus, the use of new data sources, including citizen science data may be the catalyst that forces to the surface a debate on the precise role or mandate and methods of international official statistics vis-à-vis the new dataverses and the new opportunities for the production of international statistics. Reister et al. (97) argue that this is a debate many might prefer not to have but it is hard to see how it can be avoided for much longer with the rapid changes in the data and statistics world. A subset of this debate has been shaped around the insistence that only data provided by member states should be used in the compilation of SDG indicators (98). This constraint complicates any discussion around the use of new data sources. Assuming the political barriers can be dealt with, some technical sureties will also be required to ensure that the impartiality and quality of any citizen science data are robust (97, 99). It is important that any standards applied to citizen science or any other new data sources are proportionate and do act as barriers to entry.

Finally, many citizen science data collections are local in scale. This presents some statistical challenges for extrapolating results to national level (let alone global level) – especially if several projects exist but all using different statistical or epidemiological approaches. But this challenge triggers an interesting question – what are the appropriate data to formulate local, regional or national policy? It has been argued that global level SDG indicators are better thought of as global key performance indicators, and that national data would be better suited to inform national policy (100). In assessing the potential contribution (either direct or supplementary) of citizen science data, this debate is pertinent. The analyses presented here demonstrate that there is certainly space for further thought and investigation and also a requirement for guidelines on how to incorporate citizen science data with the data sources currently being used.

### 4.1. Limitations

Our research was comprehensive and aimed to identify additional citizen science initiatives to those covered by Fraisl et al. (62). However, due to the broad scope and complex landscape of citizen science including various terminologies and definitions, one of the most important limitations of our study was that we may have left out initiatives with potential that could be featured in this study. Additionally, we only searched for initiatives that published English outputs or had English websites, which means a local scale citizen science project in another language may not have been covered by our research. Nevertheless, our purpose has not been to create an exhaustive list of all citizen science initiatives, but instead to identify additional initiatives per indicator to understand and demonstrate the potential of citizen science for monitoring and contributing to the achievement of health and well-being related global targets. Another limitation was the database search we did using SciStarter, Zooniverse and web-based searches related to citizen science projects on the topics of all the indicators covered in this study. As described in the materials and methods section, we did not search scientific databases as many local scale and community-based citizen science initiatives are not represented in peer-reviewed literature. However, a web-based search would show initiatives from both peer-reviewed and non-peer-reviewed sources, both of which are included in this study and is more inclusive in terms of covering diverse initiatives, both scientific and action-oriented. This approach was also helpful to keep the focus on the main goal of the paper, which is understanding the potential of citizen science for each health and well-being related indicator, rather than creating an exhaustive list of all or many citizen science initiatives, as mentioned above. Another limitation of this study was that we have not reviewed the methodologies, quality assurance and quality control processes of these initiatives or the resulting data sets of the projects featured in this study. Additional limitations include coverage, replicability and sustainability, as many of these initiatives are related to local contexts, and could be subject to availability of funds.

### 4.2. Recommendations

One major recommendation is that citizen science initiatives presented in this study with potential to contribute to the monitoring of health and well-being related indicators and targets can be upscaled at a national and global level by building partnerships between these initiatives, NSOs, WHO and other key stakeholders. A similar approach was implemented in a Ghana marine litter case study, where citizen science data from local level beach cleanup initiatives were integrated into the official SDG reporting of Ghana (101). These partnerships can be created in areas where there is high potential and large data gaps, as low hanging fruits, such as measuring malaria, which is the subject of indicator 3.3.3 *Malaria incidence per 1,000 population* (102) or air quality, as covered in indicators 3.9.1 *Mortality rate attributed to household and ambient air pollution* and 11.6.2 *Annual mean levels of fine particulate matter (*e.g.*, PM2.5 and PM10) in cities (population weighted)* (103, 104).

In order to integrate citizen science data into health and well-being statistics, the first step can be to identify an indicator or a topic where data gaps exist, and where citizen science data could contribute to the monitoring based on the results of this study. The second step would be to build partnerships between the NSO in a country of interest and the relevant line ministries or agencies responsible for monitoring the identified indicator, the custodian agency (or agencies), relevant citizen science initiatives, civil society and other stakeholders that have an interest in this indicator or topic, which may be academia, volunteer groups and communities. It is essential to build trust and develop a set of common goals where all the stakeholders take ownership of both the project and the results. There may be cases where the methodologies of citizen science initiatives need to be improved or adapted based on the global methodology and/or NSO requirements. In this situation, clear communication as well as intellectual and financial support should be provided to the relevant citizen science initiatives, while considering their goals and concerns, e.g., to minimize any additional burden placed on volunteers during data collection activities or to adjust protocols to fit in with their own needs and interests.

## 5. Conclusion

This paper provided a comprehensive overview of the potential of citizen science for official monitoring processes and for contributing to the achievement of health and well-being related indicators covering the SDG Global Indicator Framework and the WHO’s Triple Billion Targets. Our findings indicate that out of 58 health and well-being related indicators, citizen science has the potential to contribute to and/or complement the monitoring of 48 indicators or 83% of those identified.

Most of the examples covered in this study are from Low-and Middle-income Countries (LMICs) rather than high-resource countries. However, focusing particularly on such settings requires a detailed analysis, which is beyond the scope of this study. Future research can focus on a comparative analysis between LMICs and high-resource countries, showing the characteristics of these projects to understand the relevance of such initiatives to different contexts for their replication and upscaling potential. A specific focus on this would also be useful for demonstrating the potential of citizen science for the *leaving no one behind* principle of the SDG agenda, including the most vulnerable. Additionally, future research can build on the results of our study to focus on identifying those initiatives with the greatest potential based on the data gaps and needs of the relevant custodian agencies, in this case mostly WHO, and interested NSOs, and then focus on the data management processes and data sets of these particular projects.

## Data availability statement

The original contributions presented in the study are included in the article/Supplementary material, further inquiries can be directed to the corresponding author.

## Author contributions

DF: conceptualization, methodology, writing—original draft, investigation, writing—review and editing, project administration, resources, funding acquisition, and supervision. LS: conceptualization, methodology, writing—original draft, and writing—review and editing. DEF: Writing—review and editing. NT: writing—review and editing. SM: conceptualization, writing—review and editing, and supervision. All authors contributed to the article and approved the submitted version.

## Funding

This research was supported by the World Health Organization (WHO). The authors are responsible for the views expressed in this article and they do not necessarily represent the decisions, policy, or views of the World Health Organization. Additionally, DF and LS gratefully acknowledge funding from IIASA and the National Member Organizations that support the institute.

## Conflict of interest

The authors declare that the research was conducted in the absence of any commercial or financial relationships that could be construed as a potential conflict of interest.

## Publisher’s note

All claims expressed in this article are solely those of the authors and do not necessarily represent those of their affiliated organizations, or those of the publisher, the editors and the reviewers. Any product that may be evaluated in this article, or claim that may be made by its manufacturer, is not guaranteed or endorsed by the publisher.

## Footnotes

^1^ https://scistarter.org/

^2^ https://www.zooniverse.org/
