# Supplementary material for: Citizen science for monitoring the health and well-being related Sustainable Development Goals and the World Health Organization’s Triple Billion Targets
Source: Front Public Health. 2023 Aug 9;11:1202188. doi: 10.3389/fpubh.2023.1202188 (PMC10450341; doi:10.3389/fpubh.2023.1202188)
Supplement: Supplementary file 1 [file Table_1.docx]

**Citizen science for monitoring the health and well-being related Sustainable Development Goals and the World Health Organization's Triple Billion Targets**

Dilek Fraisl^1*^, Linda See^1^, Diana Estevez^2^, Nola Tomaska^2^, Steve MacFeely^2, 3^

^1^International Institute for Applied Systems Analysis, Laxenburg, Austria

^2^World Health Organization, Geneva, Switzerland

^3^University College Cork, Cork, Ireand

*** Correspondence:**Corresponding Author
fraisl@iiasa.ac.at

**SUPPLEMENTARY TABLE:** FULL REVIEW PRESENTING THE POTENTIAL OF CITIZEN SCIENCE FOR MONITORING THE HEALTH AND WELL-BEING RELATED INDICATORS

| **INDICATOR** | | **2020 SYSTEMATIC REVIEW RESULTS** | | **UPDATED REVIEW RESULTS FOR THE HEALTH AND WELL-BEING RELATED INDICATORS** | | | |
| --- | --- | --- | --- | --- | --- | --- | --- |
| **Target** | **Indicator** | **2020 Systematic Review Results** | **Rationale for Mapping** | **Additional Projects to 2020 Review** | **Links to References** | **Updated Rationale** | **Contribution**  **Type** |
| **Goal 1. End poverty in all its forms everywhere** | | | | | | | |
| 1.4 By 2030, ensure that all men and women, in particular the poor and the vulnerable, have equal rights to economic resources, as well as access to basic services, ownership and control over land and other forms of property, inheritance, natural resources, appropriate new technology and financial services, including microfinance | 1.4.1 Proportion of population living in households with access to basic services | **Could Contribute:**  Aqua,  Action Research for Learning Bangladesh,  Action Research for Learning Bangladesh | Among different aspects of poverty, this indicator focuses on ‘access to basic services’. Providing access to basic services such as safe drinking water, sanitation facilities, sustainable energy and mobility, housing, education, healthcare, etc., helps to improve the quality of life. Indicator 6.1.1 (Proportion of population using safely managed drinking water services) is about drinking water. Hence, the projects mapped in 6.1.1 (Aqua and Freshness of Water) could also contribute to the monitoring of this indicator. Aqua (Spain) aimed to control the quality of drinking water. Thousands of Spanish participants, particularly students, created a map with the measurements they made on water quality (chlorine, pH, flavor, smell). Freshness of Water (NL), on the microbiological stability of drinking water, enabled citizens to analyze samples from their own kitchen tap and test the water quality using test strips. In addition, Action Research for Learning Bangladesh, which was mapped as "could be used" for indicator 6.2.1 (Proportion of population using (a) safely managed sanitation services and (b) a hand-washing facility with soap and water), could also support this indicator, as it included monitoring activities through a community-based monitoring (CBM) system, where women volunteers in five selected villages in Bangladesh visited 60 neighbours each month to ask where they collected drinking water, looked at the condition of the toilet, checked that all household members were using it and asked about handwashing facilities. These aforementioned projects could provide information on the functioning of basic services so they could be used to inform some of the underlying aspects of this indicator (e.g., water, etc.). | **Could Contribute:**  WHO Snakebite Information and Data Platform,  CSIRO Energise | <https://www.who.int/teams/control-of-neglected-tropical-diseases/snakebite-envenoming/snakebite-information-and-data-platform>    <https://blog.csiro.au/lighting-up-australias-energy-future-the-csiro-energise-app-needs-you/> | In addition to the projects highlighted in the 2020 systematic review, particularly related to the healthcare aspect of this indicator, the WHO initiative "Snakebite Information and Data Platform" can contribute to the monitoring of this indicator. Among different aspects of poverty, this indicator focuses on ‘access to basic services’. Providing access to basic services such as safe drinking water, sanitation facilities, sustainable energy and mobility, housing, education, healthcare, etc., helps to improve the quality of life. WHO's Snakebite Information and Data Platform aims to stimulate work towards achieving the related global target to “halve the number of deaths and disability due to snakebite envenoming by 2030”. Additionally, the platform seeks to advance surveillance and contribute to related epidemiological documentation and data, which countries can use to compile statistics for SDG monitoring and beyond. This can allow for more effective sharing of resources and coordination of prevention efforts while also improving antivenom distribution and mapping. The platform allows the public to participate and contribute by sharing photos of what they think are venomous snakes along with their location data. These data can assist WHO experts to refine and enhance the distribution maps and provide communities with more reliable and accurate information as a source of real-time data through citizen science. The portal also shows the locations of antivenom treatment facilities, enabling prompt patient access. Hence, the project provides an example of how citizen science data along with other information can enable access to healthcare.  Additionally, because the indicator has a component related to "access to clean fuels and technology" (also covered as part of indicator 7.1.2), CSIRO Energise can also contribute to the health related aspect of this indicator. CSIRO Energise, implemented in Australia, has helped scientists to better understand how households across the country use, generate and interact with energy. The data provided by citizen scientists has been crucial in order to conduct research towards a more secure, reliable and sustainable energy future for Australia. The app produced data on household energy use including on appliance usage and energy bills, through to the emergence of new technologies. | Supplementary |
| 1.5 By 2030, build the resilience of the poor and those in vulnerable situations and reduce their exposure and vulnerability to climate-related extreme events and other economic, social and environmental shocks and disasters | 1.5.1 Number of deaths, missing persons and directly affected persons attributed to disasters per 100,000 population | **Could Contribute:**  Haze Gazer | Haze Gazer is a crisis analysis and visualization tool that produces real-time situational data from diverse data sources, including: open data in the form of fire hotspot information from satellites and baseline information on population density and distribution, citizen science and citizen generated data such as from the national complaint system in Indonesia called LAPOR, citizen journalism from videos uploaded to an online news channel and social media data such as online video channels, like YouTube and Instagram. | **Could Contribute:**  Picture Pile,  Humanitarian OpenStreetMap,  Missing Maps,  University of Oxford-Zooniverse-Rescue Global partnership for Hurricane Irma | <https://www.isprs-ann-photogramm-remote-sens-spatial-inf-sci.net/IV-4/27/2018/>   <https://www.sciencedirect.com/science/article/pii/S1462901121003208?via%3Dihub>   <https://www.hotosm.org/disaster-services/project_activations>   <https://www.missingmaps.org/>   <https://www.ox.ac.uk/news/2017-09-20-artificial-intelligence-citizen-science-and-disaster-response-combine-help-victims>   <https://www.nature.com/articles/515321a>   <https://www.nature.com/articles/nature.2013.14186>   <https://www.frontiersin.org/articles/10.3389/feart.2019.00226/full>   <https://www.sciencedirect.com/science/article/pii/S1462901121003208> | The metadata for this indicator refers to the Technical Guidance for Monitoring and Reporting on Progress in Achieving the Global Targets of the Sendai Framework for Disaster Risk Reduction for the detailed methodology. According to this technical guidance, indicator 1.5.1 is linked to Target A "Substantially reduce global disaster mortality by 2030, aiming to lower average per 100,000 global mortality between 2020-2030 compared to 2005-2015" and Target B "Substantially reduce the number of affected people globally by 2030, aiming to lower the average global figure per 100,000 between 2020-2030 compared to 2005-2015" of the Sendai Framework. Under Target B, five indicators are recommended for measurement. Among those, B-3 indicator is the "number of people whose damaged dwellings were attributed to disasters", while B-4 is about the "number of people whose destroyed dwellings were attributed to disasters". For the computation of B-3 and B-4, "number of dwellings damaged" and "number of dwellings destroyed" are needed. Although the methodology suggests that these two indicators are measured in-situ, their sub-indicators "number of dwellings/houses damaged attributed to disasters" and "number of dwellings/housed destroyed attributed to disasters" could benefit from the input that Picture Pile could offer. Picture Pile is a web and mobile application designed for rapid image classification from satellite images and orthophotos, geo-tagged photographs, drone imagery, etc. For the post disaster damage assessment, Picture Pile can be used for the classification of satellite images to identify damaged buildings after a disaster. Such a campaign has already been implemented after Hurricane Matthew in 2017. The published results are in the "links to references" column for 1.5.1 in this Supplementary Material.  Additionally, in Humanitarian OpenStreetMap, volunteers create maps using satellite and drone imagery for rapid response by aid organizations to reach affected areas and those in need after a disaster.  Missing Maps is another citizen science project that aims to create accessible map data where humanitarian organizations are operating. They aim to grow a global mapper community to support those living in places at risk through the use of OpenStreetMap and contributions from community volunteers.  As part of the University of Oxford-Zooniverse-Rescue Global project for Hurricane Irma, through volunteer classification of satellite images before and after the hurricane, data on damaged buildings, areas of flooding, blocked roads, and temporary settlements that may indicate displaced or homeless populations were collected. Additional links that include more examples and evidence of how citizen science data could be used to monitor this indicator are provided in the "links to references" column for indicator 1.5.1 in this Supplementary Material. | Direct and Supplementary |
| 1.a Ensure significant mobilization of resources from a variety of sources, including through enhanced development cooperation, in order to provide adequate and predictable means for developing countries, in particular least developed countries, to implement programmes and policies to end poverty in all its dimensions | 1.a.2 Proportion of total government spending on essential services (education, health and social protection) | **No alignment** identified with any existing or past citizen science project. | N/A | **No alignment** | N/A | N/A | N/A |
| **Goal 2. End hunger, achieve food security and improved nutrition and promote sustainable agriculture** | | | | | | | |
| 2.2 By 2030, end all forms of malnutrition, including achieving, by 2025, the internationally agreed targets on stunting and wasting in children under 5 years of age, and address the nutritional needs of adolescent girls, pregnant and lactating women and older persons | 2.2.1 Prevalence of stunting (height for age <-2 standard deviation from the median of the World Health Organization (WHO) Child Growth Standards) among children under 5 years of age | **No alignment** identified with any existing or past citizen science project. | N/A | **No alignment** | N/A | No alignment identified with any existing citizen science project. However, there is certainly scope for citizen science approaches to be implemented to monitor this indicator, as reported in the justification for indicator 2.2.2 below. Similar initatives as in 2.2.2 could be adjusted to gather complementary data for this indicator. | N/A |
|  | 2.2.2 Prevalence of malnutrition (weight for height >+2 or <-2 standard deviation from the median of the WHO Child Growth Standards) among children under 5 years of age, by type (wasting and overweight) | **No alignment** identified with any existing or past citizen science project. | N/A | **Could Contribute:**  BigO,  Community-based growth monitoring model in a semi-urban community in South Africa,  EPODE | <https://bigoprogram.eu/big-data-against-childhood-obesity/>  <https://mhealth.jmir.org/2021/7/e26290>  <https://ieeexplore.ieee.org/document/9175361>  <https://journals.sagepub.com/doi/pdf/10.1177/156482650302400405>  <https://www.eufic.org/en/healthy-living/article/preventing-childhood-obesity-through-community-based-initiatives>  <https://onlinelibrary.wiley.com/doi/full/10.1111/j.1467-789X.2011.00950.x> | This indicator has 2 subindicators. Indicator 2.2.2a is about "prevalence of overweight (weight for height >+2 standard deviation from the median of the World Health Organization (WHO) Child Growth Standards) among children under 5 years of age" and 2.2.2b is about "prevalence of wasting (weight for height <-2 standard deviation from the median of the World Health Organization (WHO) Child Growth Standards) among children under 5 years of age".  For 2.2.2a, even though the target group is school aged children and not under 5, BigO could probably be adapted to contribute to the monitoring of this indicator, as it collects and analyses anonymous data on children's behavioural patterns and their living environment. BigO uses advanced analytics to gather information about the local factors at play and how they affect childhood obesity across Europe. In BigO, school aged children provide data about their behavioural patterns and local environment, using the myBigOapp. To better understand how behaviours and the environment affect the prevalence of obesity, these data are anonymized and utilised to develop complex statistical models. These anonymous data are used to forecast how policy changes or advancements may affect obesity rates, advance understanding of the underlying causes of childhood obesity, and guide the design of regional initiatives for both prevention and treatment.  As part of a research study conducted in 2003, a community-based growth monitoring technique in a semi-urban settlement in South Africa was used. The goal was to determine whether a community-based growth monitoring methodology can be used to address the problems with pre-school aged children's health and nutrition surveillance. Baseline surveys and community-based interactive workshops were organised in order to carry out the study. Eleven women were then trained to lead the community-based growth monitoring project. The findings showed that community involvement and mobilization can improve health and nutrition surveillance of preschool children's growth while expanding the scope. Even though this was designed as a study rather than an ongoing project, the results are important in terms of demonstrating the potential of such initiatives in understanding malnutrition among preschool aged children.  EPODE, “Ensemble Prevenons l’Obesité Des Enfants”, meaning “Together let’s prevent childhood obesity” was a program that was implemented in two communities in northern France. The aim was to empower communities to combat and prevent childhood obesity. Primary EPODE target groups were all children aged 0–12 years. As part of the program, a local steering committee including local stakeholders in schools, extracurricular organisations and social networks of associations was mobilized to place the community at the heart of the system. Community-based initatives like EPODE can help mobilize local actions to address the global issue of child obesity or malnutrition, and support both monitoring of this indicator and the implementation of the relevant target. | Supplementary |
|  | 2.2.3 Prevalence of anaemia in women aged 15 to 49 years, by pregnancy status (percentage) | This indicator was included in the SDG Indicator Framework after the 2020 systematic review was completed. | New indicator/Not mapped in 2020. | **Could Contribute:**  Reduce Maternal Anemia in Nepal | <http://www.a2zproject.org/pdf/ReducingAnemiaNepal.pdf> | As part of the the Iron Intensification Project (IIP) that covered 70 districts in Nepal, female community health volunteers (FCHVs), after receiving training, informed the community about the importance of iron supplementation and related interventions through various social groups. Then they distributed iron-folic acid (IFA) to pregnant woman. Before the program, IFA was available only through health facilities. Therefore, involving the FCHVs significantly enhanced community access to these supplements. Where necessary, the volunteers visited the pregnant women as frequently as possible to make sure the pills were taken and to discuss the benefits of doing so. Results were successful. Maternal anaemia prevalence in the country decreased significantly between 1998 and 2006: from 68% to 36% in women of reproductive age, and from 75% to 42% in pregnant women. One of the key factors for success was the community-based delivery of committed and reputable FCHVs. They were trusted by the community and their activities have helped to build community trust in the health system. Such community-based initiatives cannot only provide data for this indicator, but also result in achieving the relevant target. | Supplementary |
| **Goal 3. Ensure healthy lives and promote well-being for all at all ages** | | | | | | | |
| 3.1 By 2030, reduce the global maternal mortality ratio to less than 70 per 100,000 live births | 3.1.1 Maternal mortality ratio | **Could Contribute:** Saving Mothers Giving Life (SMGL) Uganda, Community-based surveillance of maternal deaths in rural Ghana | Uganda lacks recent, accurate population data. However, there are village health teams (VHTs) in Uganda, which typically include 5-6 community volunteers to provide maternal, newborn and child services. They are the bridge between the community and health services. As part of the Saving Mothers Giving Life (SMGL) iniatives, about 4000 VHTs, one per village, were trained to provide preventive maternal and child health services and to report on the deaths of women of reproductive age. Hence, the SMGL project could be mapped as "could be used". Another example is the community-based surveillance of maternal deaths in rural Ghana, where volunteers are engaged to conduct a survey on maternal deaths after training. | **Could Contribute:**  POWERMOM | <https://www.hcinnovationgroup.com/population-health-management/mobile-health-mhealth/news/21249309/scripps-launches-maternal-health-research-consortium>    <https://www.cambridge.org/core/journals/journal-of-clinical-and-translational-science/article/pregnancy-health-in-powermom-participants-living-in-rural-versus-urban-zip-codes/049D61ADB9F1F8E084B57300C23E4E15> | In addition to the projects highlighted in the 2020 systematic review results, projects like US based POWERMOM can help produce actionable results related to maternal mortality, especially among underrepresented groups. Even though the aim is not measuring maternal mortality, POWERMOM gathered data on the health of pregnant women, which could provide useful insights for the indicator and support the relevant target. Led by a CA based Scripps Research Digital Trials Center and utilizing digital and mobile technology, the project intended to enhance maternal and fetal health outcomes. The objective was to create the largest, most diverse community of expectant women while also gathering important health data to support new mothers. The PowerMom team created an app-based research platform to accomplish this, enabling expectant mothers to share health information via surveys, electronic health records, and wearables like fitness trackers and smartwatches. By enabling participants to enroll and participate in the study remotely, the scientists intended to remove some of the obstacles that exist for traditional clinic-based studies, which have historically lacked diversity. | Supplementary |
|  | 3.1.2 Proportion of births attended by skilled health personnel | **Could Contribute:** Saving Mothers Giving Life (SMGL) | Community-based monitoring projects, such as SMGL (mentioned in 3.1.1), could be used for the monitoring of births attended by skilled personnel, which are mentioned in SMGL. The main source of data for this indicator is household surveys. This makes the measurement of mortality in near real-time or more frequently very difficult. In addition, women who give birth at home may be excluded from the calculations when the facility data are used. Hence, data from SMGL can provide supplementary information for the monitoring of this indicator, and it could support the verification of the data from traditional sources such as household surveys and administrative records. | **Could Contribute:**  Saving Mothers Giving Life (SMGL) | <https://www.cdc.gov/reproductivehealth/global/programs/saving-mothers-giving-life/index.htm#:~:text=The%20Saving%20Mothers%2C%20Giving%20Life,maternal%20and%20newborn%20deaths%20occur>.  <https://www.ghspjournal.org/content/7/Supplement_1> | SMGL, which was mapped as could contribute in the 2020 systematic review, focusses also on "skilled caregivers at birth" in its multi-system approach. Other components of this approach included (i) safe facilities and hospitals for delivery, (ii) supplies for and provision of basic and emergency obstetric services, (iii) systems for communication, referral, and transportation that are available 24 hours a day, 7 days a week, and (iv) quality data, surveillance, monitoring, and evaluation. | Supplementary |
| 3.2 By 2030, end preventable deaths of newborns and children under 5 years of age, with all countries aiming to reduce neonatal mortality to at least as low as 12 per 1,000 live births and under-5 mortality to at least as low as 25 per 1,000 live births | 3.2.1 Under-5 mortality rate | **Could Contribute:** Saving Mothers Giving Life (SMGL), Citizen Report Cards India | SMGL (see 3.1.1 & 3.1.2) “could be used” to collect data on under 5 mortality as well. In addition, Citizen Report Cards India could be used for monitoring this indicator. Citizen Report Cards, within the framework of India’s National Rural Health Mission (NRHM), was launched in 2005, and was due to end in 2012. It aimed to improve the quality of health care in 18 states with the poorest health indicators through implementation of a health systems strengthening approach. As one of the aims of the initiative was validating the data collected in official ways (i.e., through Auxillary Nurse Midwifes, Anganwadi workers -which are volunteer child health and nutrition workers- and other functionaries of the public health system) using community monitoring, Citizen Report Cards India can be mapped as “could contribute”. | **Could Contribute:**  CARE Group Model | <https://systematicreviewsjournal.biomedcentral.com/articles/10.1186/s13643-020-01497-1>  <https://www.spring-nutrition.org/publications/case-studies/care-group-reducing-malnutrition-and-child-deaths-mozambique>  <https://bmcpublichealth.biomedcentral.com/articles/10.1186/s12889-015-2187-2> | In addition to the projects highlighted in the 2020 systematic review, the CARE Group Model, adopted in Mozambique in 1997 and used in about 30 countries since then, can support the monitoring of this indicator, as well as supporting action. A Care Group is a group of 10-15 volunteers and community-based health educators, who regularly meet together with project staff for training and supervision. Each volunteer is in charge of regularly visiting 10-15 of their neighbors, sharing what they have learned and encouraging behavioural change at the household level. Care Groups create a multiplicative effect that reaches every beneficiary household in a far reaching way. A community health information system that tracks new pregnancies, births, and deaths discovered during home visits is also made possible by these groups. The Care Group Model may offer a viable strategy for amplifying critical child survival interventions. | Supplementary |
|  | 3.2.2 Neonatal mortality rate | **Could Contribute:** Saving Mothers Giving Life (SMGL), Citizen Report Cards India | Same rationale as 3.2.1. | **Could Contribute:**  CARE Group Model | See the links in 3.2.1. | In addition to the projects highlighted in the 2020 systematic review, CARE Group approaches as described in 3.2.1 can provide a contribution to related action and monitoring activities concerning this indicator (see the 3.2.1 justification on the CARE Group Model). | Supplementary |
| 3.3 By 2030, end the epidemics of AIDS, tuberculosis, malaria and neglected tropical diseases and combat hepatitis, water-borne diseases and other communicable diseases | 3.3.1 Number of new HIV infections per 1,000 uninfected population, by sex, age and key populations | **Could Contribute:** Engage-TB, OneImpact | Engage-TB (tuberculosis) is a WHO-recommended approach for integrating community-based TB activities into NGO and CSO activities. WHO-supported pilot projects have implemented the ENGAGE-TB approach in five countries (Democratic Republic of Congo, Ethiopia, Kenya, South Africa, and Tanzania) since early 2012. A monitoring sheet was used to collect data on TB. Community health workers and community volunteers carried out community-based TB activities such as screening and referral. As Engage-TB "could contribute" data on TB and related diseases such as HIV, it could also be used for monitoring of this HIV indicator as well. One Impact, an app designed to support community-based monitoring of TB response, could support this indicator by being expanded to capture data on HIV as well. These approaches could provide supplementary information that may also be useful for geospatial disaggregation. | **Could Contribute:**  Ritshidze Project,  Community Treatment Observatory (CTO) | <https://www.unaids.org/sites/default/files/media_asset/establishing-community-led-monitoring-hiv-services_en.pdf>  <https://ritshidze.org.za/wp-content/uploads/2020/11/Ritshidze-Activist-Guide-2020-1.pdf>  <https://ritshidze.org.za/the-model/>  <https://itpcglobal.org/wp-content/uploads/2019/02/ITPC-CTO-Model-Full-Eng.pdf>  <https://link.springer.com/article/10.1007/s11904-020-00521-2> | In addition to the projects highlighted in the 2020 systematic review, the Ritshidze (Saving our lives) Project can contribute to this indicator. Ritshidze is a community led monitoring model that has been developed to give communities the tools and methods for monitoring and assessing the quality of HIV, TB and other health services provided at clinics and elevating issues with performance to appropriate decision makers in order to advocate for change. The project tools gather data both at the clinic and in the community levels. The “facility-based” monitoring records observations of patients and healthcare providers. Through door-to-door interaction, one-on-one interviews, and informal focus groups, community organisations collect data for the community-based monitoring component of the project directly from community members. Even though the project is not developed to identify the number of new HIV infections, it can serve the achievement of the target, and with modifications and if expanded, could provide useful supplementary information for the indicator. The project is covering nearly half of the population living with HIV in South Africa (the Ritshidze Project’s background and reports are available at https://ritshidze.org.za/).  Additionally, the Community Treatment Observatory (CTO) Model is a model that collects and analyzes qualitative and quantitative data systematically and periodically. These data are used to track trends in HIV treatment and care, and to inform targeted interventions that will raise the quality of HIV services. In a CTO, an organized group of community members such as a network of HIV-positive individuals gathers information on various aspects of HIV prevention, testing, care, and treatment services. One of the results of the model is that, in Sierra Leone, HIV testing increased among men who have sex with men (85%), female sex workers (100%), people who inject drugs (96%), pregnant women (90%), and young people (90%) in a year of implementation (Baptiste et al, 2020). Such community-based monitoring intiaitives are not only helpful for better monitoring of HIV but also in terms of stimulating awareness and action. | Supplementary |
|  | 3.3.2 Tuberculosis incidence per 100,000 population | **Could Contribute:** OneImpact, Engage-TB | OneImpact could be used for monitoring this indicator. It offers a platform that allows community organizations to track, monitor and respond to challenges on TB services. OneImpact also generates data to improve local and national TB responses. It tracks the issues reported, maps the frequency and location of issues, and designs and circulates surveys, among others. Engage-TB can also be mapped as "could be used" for this indicator. It is a WHO-recommended approach for integrating community-based TB activities into the activities of NGOs and CSOs. These approaches could provide supplementary information that may also be useful for geospatial disaggregation. | **Could Contribute:**  The Ritshidze Project,  Community Treatment Observatory (CTO) | See the links in 3.3.1  <https://www.who.int/publications/i/item/9789240061729> | In addition to the projects highlighted in the 2020 systematic review, the Ritshidze (Saving our lives) Project, and the Community Treatment Observatory (CTO) Model are not only helpful for better monitoring of this indicator but also in terms of stimulating related awareness and action (see the justification in 3.3.1 above).  The projects mapped in 3.3.1 and 3.3.2 are the same, as they can be expanded to provide additional information and aspects on both tuberculosis and HIV even beyond the monitoring needs such as policy response and awareness raising. For example, according to the WHO, deaths from tuberculosis among HIV-positive people are officially classified as deaths caused by HIV/AIDS, with tuberculosis as a contributory cause (see the WHO 2022 Global Tuberculosis report in the previous column). | Supplementary |
|  | 3.3.3 Malaria incidence per 1,000 population | **Could Contribute:** MAHEFA (MA-lagasy HE-althy FA-milies), MAHEFA Miaraka, Community-based Surveillance System (CBSS) - Rural Cambodia, Humanitarian OpenStreetMap Malaria Elimination Program Botswana,  Global Mosquito Alert,  Mosquito Alert | MAHEFA (MA-lagasy HE-althy FA-milies) is a community-based health project by USAID and the Madagascar Ministry of Public Health. The program has trained thousands of volunteers, who are people chosen by their communities, to provide basic health services, to identify serious cases and to get people the help they need. Community health volunteers in Analalava, for instance, were tasked with many services that were previously not available (e.g., promoting good health practices and hygiene, providing family planning services, and diagnosing and treating simple cases of malaria, diarrhoea and pneumonia for children under age 5, raising childhood immunization, etc.). Therefore, MAHEFA could be used for monitoring this indicator. The Community-based Surveillance System (CBSS) - Rural Cambodia initiative could be used for monitoring this indicator as well. It is a WHO initiative, where WHO is the custodian for this indicator. Events reported by the CBSS were identified through discussion with health staff and Village Health Volunteers (VHVs) based on their public health importance, severity and potential for an outbreak as well as the existence of a control programme. This included malaria, chronic cough, acute severe diarrhoea, measles and haemorrhagic fever, and births and deaths. Data on these events were collected by VHVs and reported to data collation and analysis teams based in health centres. The Humanitarian OpenStreetMap (HOT OSM) Malaria Elimination Program in Botswana was adapted to understand people’s exposure to mosquitos through locating houses and field surveys to check if these houses have complete walls and roofs. Additionally, Mosquito Alert and Global Mosquito Alert collect data on mosquito populations and their possible breeding sites, which could provide supplementary data for the indicator. | **Could Contribute:**  Malaria Mosquito Surveillance in Ruhuha Rwanda, Globe Mosquito Habitat Mapper | <https://malariajournal.biomedcentral.com/articles/10.1186/s12936-021-03989-4>  <https://doi.org/10.1029/2021GH000436>  <https://doi.org/10.1186/s12936-020-03349-8> | In addition to the projects highlighted in the 2020 systematic review, in the Malaria Mosquito Surveillance program in Ruhuha, Rwanda, a 1-year citizen science program for malaria mosquito surveillance was implemented in five villages of the Ruhuha sector, Rwanda. As part of the program, citizens reported monthly data on mosquitoes they collected using handmade carbon-dioxide baited traps. Additionally, they reported mosquito nuisance experienced as well as the number of confirmed malaria cases in their household.  Globe's Mosquito Habitat Mapper project allows citizen scientists to report the mosquito larval habitats they identify using the GLOBE Mosquito Habitat Mapper tool. These data can complement the climate, weather, and land cover data obtained from satellite measurements by scientists who develop risk models for mosquito-borne diseases. The program provides the opportunity to obtain the volume, velocity and variety of data needed to fight the threat of vector-borne diseases, especially in under-resourced communities. | Direct and Supplementary |
|  | 3.3.4 Hepatitis B incidence per 100,000 population | **Could Contribute:** OneImpact, Engage-TB | The Engage TB and One Impact initiatives could be extended to capture data on Hepatitis B and hence they "could be used" for monitoring this indicator as well. See the rationale for 3.3.2 for more information. | **Could Contribute:**  Community Treatment Observatory (CTO) Model | <https://www.amfar.org/treat-asia/cto-analysis-2022/> | The Community Treatment Observatory (CTO) model, as a broad approach, can support this indicator, in addition to indicators 3.3.1 and 3.3.2 above, and mobilize action for the achievement of the relevant target (see the justification in 3.3.1 and 3.3.2). | Supplementary |
|  | 3.3.5 Number of people requiring interventions against neglected tropical diseases | **Could Contribute:**  Community-based Surveilance System (CBSS) - Rural Cambodia | Community-based Surveilance System (CBSS) - Rural Cambodia could be used for monitoring this indicator. This particular case mainly focused on diseases such as malaria, chronic cough, acute severe diarrhoea, measles and haemorrhagic fever, as well as births and deaths due to the adaptation to case definitions used at the relevant health center. However, the idea was targeting all common diseases and vital events as previous similar programs that focussed on a single disease did not maximize the value of resources put in for the initiative so it reported on any disease and event that could be a potential outbreak. Hence, it could also help the monitoring of neglected tropical diseases. The way it worked: the events to be reported by the CBSS were identified through discussion with health staff and VHVs based on their public health importance, severity and potential for an outbreak as well as the existence of a control programme. Data on these events were collected by VHVs and reported to data collation and analysis teams based in health centres. This shows that citizen science could feed into epidemiology models, and also help in estimating disease prevalence. | **Could Contribute:**  Snakebite Information and Data Platform | <https://www.who.int/teams/control-of-neglected-tropical-diseases/snakebite-envenoming/snakebite-information-and-data-platform> | WHO's own initiative "Snakebite Information and Data Platform", as presented in indicator 1.4.1 above, can provide useful information to support this indicator, as well as the achievement of the relevant target. The platform aims to stimulate work towards achieving the related global target to “halve the number of deaths and disability due to snakebite envenoming by 2030”. Additionally, the platform seeks to advance surveillance and contribute to related epidemiological documentation and data, which countries can use to compile statistics for SDG monitoring and beyond. This can allow more effective sharing of resources and coordination of prevention efforts while also improving antivenom distribution and mapping. The platform allows the public to participate and contribute by sharing photos of what they think are venomous snakes along with their location data. These data can assist WHO experts to refine and enhance the distribution maps and provide communities with more reliable and accurate information as a source of real-time data through citizen science. The portal also shows the locations of antivenom treatment facilities, enabling prompt citizen access. Hence, the project provides an example of how citizen science data along with other information can enable access to healthcare. | Direct |
| 3.4 By 2030, reduce by one third premature mortality from non-communicable diseases through prevention and treatment and promote mental health and well-being | 3.4.1 Mortality rate attributed to cardiovascular disease, cancer, diabetes or chronic respiratory disease | **No alignment** identified with any existing or past citizen science project. | N/A | **Could Contribute:**  Community-Driven Citizen Science Approach to Explore Cardiovascular Disease Risk Perception and  Develop Prevention Advocacy Strategies in Sub-Saharan Africa | <https://researchinvolvement.biomedcentral.com/articles/10.1186/s40900-020-00246-x>  <https://doi.org/10.3389/fitd.2021.752357>  <https://www.cebha-plus.org/wp-research> | Community-Driven Citizen Science Approach to Explore Cardiovascular Disease Risk Perception and Develop Prevention Advocacy Strategies is a study implemented in rural and urban Sub-Saharan Africa. It aimed to offer a protocol outlining the planned processes for carrying out community-driven citizen science research to look into perceptions of cardiovascular disease risk and to create community-specific advocacy and prevention plans. The community leaders were recruited by their communities and were trained as citizen scientists as part of the program to interview and understand how the residents in their rural and urban communities perceive, analyze, and communicate threat and health risk associated with heart-disease. Additionally, the citizen scientists were trained by the country local research teams on how to use the project app to gather data and provide information and understanding to support disease prevention. To jointly create effective prevention programs for local communities, the research team and citizen scientists engaged the community and stakeholders and held consultations with them. The initiative is part of the CEBHA+ project funded by the German Federal Ministry of Research and Education. Because the project also addresses diabetes and hypertension, in addition to cardiovacular disease, it may not directly support the monitoring of related mortality rate in its current state, but it could provide additional information to conceptualize the indicator and could contribute to the achievement of the relevant target through its action-oriented focus. | Supplementary |
|  | 3.4.2 Suicide mortality rate | **No alignment** identified with any existing or past citizen science project. | N/A | **Could Contribute:**  Community-Based Surveillance and Case Management for Suicide Prevention: An American Indian Tribally Initiated System | <https://www.ncbi.nlm.nih.gov/pmc/articles/PMC4035881/> | Community-Based Surveillance and Case Management for Suicide Prevention: An American Indian Tribally Initiated System is an initiative that was launched to address high rates of suicide in youths by the White Mountain Apache Tribe (WMAT, or Apache) with technical support from the John Hopkins University Center for American Indian Health (JHU). As part of the project, a community-based suicide surveillance system was developed. The system featured a community-based reporting system for suicide behaviour, involvement and referral of those who were affected by it, and the creation of prevention tactics that took into account trends of suicidal behaviour on the reservation. In addition to the reports by those who saw or learned about a person engaging in self-destructive behaviour through medical, educational, and social service personnel, first responders, religious figures, family members, etc., the suicide death was also documented as part of the project through police reports.The strengths of the system were: (i) it can collect accurate, real-time data to identify trends and suicidal behaviour outside of clinical settings and coroner-level data to improve prevention tactics and action; (ii) its comprehensive system, which includes suicide ideation, attempts, and deaths as well as nonsuicidal self-injury (NSSI) and binge drinking, enables investigation of the relationships among behaviours along the spectrum of self-injury and offers an in-depth comprehension of patterns of suicidal behavior; (iii) offers a unique form of collective consent to detect and aid people who are engaging in suicidal and self-destructive behaviors —a population that frequently does not seek help and finds it difficult to connect with treatment— as part of a comprehensive public health response, among others. The project is prevention and action focused, rather than monitoring suicide mortality, but it can help gather the data to conceptualize this SDG indicator and contribute to the relevant target. | Supplementary |
| 3.5 Strengthen the prevention and treatment of substance abuse, including narcotic drug abuse and harmful use of alcohol | 3.5.1 Coverage of treatment interventions (pharmacological psychosocial and rehabilitation and aftercare services) for substance use disorders | **Could Contribute:**  A-CHESS,  OD Help | Although some clarifications are needed on the indicator description, the A-CHESS and OD Help initiatives can be mapped as could contribute as they can provide supplementary information for this indicator. A-CHESS collects data on patient demographics, a patient’s level of self-efficacy and coping style, the therapeutic goals and care plan, the patient's progress, etc. In addition, “coverage of treatment interventions” include pharmacological, psychosocial and rehabilitation, and aftercare services. OD Help is a crowdsourcing project to find the fastest way to obtain anti-overdose drugs to potential victims by mobilizing citizens, and often a driver is closer than the nearest ambulance. | **Could Contribute:**  A-CHESS | <https://www.ncbi.nlm.nih.gov/pmc/articles/PMC3536059/> | The indicator is defined in the metadata as the coverage of treatment interventions for substance use disorders, which is further defined as “the number of people who received treatment in a year divided by the total number of people with substance use disorders in the same year”. This indicator is disaggregated by two broad groups of psychoactive substances: (1) drugs, (2) alcohol and other psychoactive substances. One of the data sources for the indicator, as highligted in the metadata is surveys among people using substances, which can be supported through citizen science approaches as well. The project A-CHESS, as highlighted in the 2020 systematic review, which collects information on patient demographics, a patient's level of self-efficacy and ways of coping, the therapeutic objectives and treatment plan, and their progress can offer helpful background to conceptualize the indicator in terms of a substance use disorder. | Supplementary |
|  | 3.5.2 Alcohol per capita consumption (aged 15 years and older) within a calendar year in litres of pure alcohol | **No alignment** identified with any existing or past citizen science project. | N/A | **Could Contribute:**  Citizen science for public health through alcohol advertising | <https://academic.oup.com/heapro/advance-article/doi/10.1093/heapro/daab139/6361023?guestAccessKey=d8aeda30-b89f-49c8-8a92-9c72019ff75b&fbclid=IwAR3w7Ju0rk0GIuFB5OP8pfnX9UKfv8Rjl4YsgCxGDYCdyIp5aGUD32NcYEw> | In a citizen science project that aimed to understand the feasibility of using citizen science to identify the impact of alcohol advertising on Australian women through a breast cancer prevention project, the recruited citizen scientists first completed demographic and behavioural questions via an online survey. Then, they recorded and classified web advertisements for alcohol. As part of the survey responding process, citizen scientists were asked questions such as if they ever consumed alcohol, their preferred type of drink, places where they consume alcohol, volume and style of alcohol consumption, the number of days alcohol was consumed per month, the average and maximum number of drinks consumed per day and style of drinking, i.e., binge, occasional, weekend or everyday drinking, among others. Although the data collected through the project would not directly contribute to indicator monitoring, it can be useful for understanding alcohol consumption among participants, but also for engaging with women in alcohol advertising and breast cancer prevention research, and raise awareness on the topic among women. | Supplementary |
| 3.6 By 2020, halve the number of global deaths and injuries from road traffic accidents | 3.6.1 Death rate due to road traffic injuries | **No alignment** identified with any existing or past citizen science project. | N/A | **Could Contribute:**  Development and Implementation of Integrated Road Traffic Injuries Surveillance – India (IRIS-India), Canadian Aboriginal communities: a framework for injury surveillance,  Bike Barometer,  SimRa: Safety in Bicycle Traffic | <https://apps.who.int/iris/bitstream/handle/10665/43271/9241546751_eng.pdf>  <https://www.ncbi.nlm.nih.gov/pmc/articles/PMC7163273/>  <https://academic.oup.com/heapro/article/16/2/169/653447>  <https://doi.org/10.1016/j.cstp.2022.05.013>  <https://www.tu.berlin/en/topics/knowledge-exchange/2020/mai/safety-in-the-bicycle-saddle> | Even though the projects and the justification presented here are not related to measuring death rate due to road traffic injuries, they can still provide useful insight for the indicator as they are about traffic injuries.  The manual "Road traffic injury prevention training manual" by WHO and the Indian Institute of Technology highlights that gathering data on road traffic injuries through conducting community-based surveys is one approach to understand the extent of the problem. This is due to the fact that some injured people, for a number of reasons, do not make it to hospitals, in which case they will not be recorded in injury surveillance systems of hospitals. For example, in one study, an electronic-based comprehensive and Integrated Road Traffic Injuries (RTI) Surveillance system was established. One trauma centre, one private hospital and a community of 10000-population in urban areas were part of the system. In rural areas, a district hospital, a private nursing home and two sub-centres of different primary health centres were included in the surveillance. In the study, active surveillance was placed in communities to track missing cases. The results show that a model of surveillance including both passive and active surveillance, which involves community members, is helpful to cover the greatest number of injuries.  Additionally, in a study that aimed to develop a framework for injury surveillance in Canadian Aboriginal communities, an injury surveillance framework that would be culturally relevant, that is ‘acceptable and owned’ by the community, and would meet the specific requirements for injury data and data collection methods, was developed. The injuries in the study included falls, motor vehicle collisions, and injuries among young children, among others. Basic analysis and report generation at the community level were made possible by the approach.  Citizen science projects related to road safety, even though not related to monitoring the death rate, can provide additional supplementary information to contextualize road injuries and safety issues. For example, the Bike Barometer project from Flanders, Belgium, which involved 1,256 teenagers from 31 schools digitizing 5657 km of roads and evaluating 3,750 km of those for cycling friendliness and safety, can provide insights into safety conditions of roads in Flanders (and for particular school neighborhoods) and is highly relevant for local decision-making. Similar data about nearby crashes and the primary routes of bicycle traffic are gathered in the Berlin initiative "SimRa -Safety in Bicycle Traffic" using a smartphone app. Following that, proposals for changes in urban planning and transport policy can be made using these data. Even though the indicator aims to measure death rate, the above-mentioned and other similar projects cannot only provide useful data on potential injuries or deaths, but can also help to mobilize action toward the achievement of the target. | Supplementary |
| 3.7 By 2030, ensure universal access to sexual and reproductive health-care services, including for family planning, information and education, and the integration of reproductive health into national strategies and programmes | 3.7.1 Proportion of women of reproductive age (aged 15–49 years) who have their need for family planning satisfied with modern methods | **Could Contribute:**  CBM on Quality of Care in Family Planning Services - India,  MAHEFA (MA-lagasy HE-althy FA-milies), MAHEFA Miaraka | Community-based Monitoring (CBM) on Quality of Care in Family Planning Services India can be mapped as "could contribute". The main goal of the project was to identify the quality of care in family planning. Data collected throughout the community scorecard process, before and after, provided information on women of reproductive age who have need of family planning satisfied with modern methods (as the indicator requires). MAHEFA (MA-lagasy HE-althy FA-milies) can also be mapped as “could be used”, because it provides family planning services (see 3.3.3). | **Could Contribute:**  Reproductive Health Observatories (Guatemala) | <https://www.healthpolicyproject.com/pubs/449_PSocialAccountabilityReportFINALEC.pdf>  <https://osarguatemala.org/embarazos-y-registro-de-nacimientos-2021/> | In additon to the projects highlighted in the 2020 systematic review, in Guatemala, OSARs (Observatorios en Salud Reproductiva, or Reproductive Health Observatories), independent civil-society-led bodies, were established at both the national and local levels through a memorandum of understanding with the government and supported by donor funding. They focus on accountability, monitoring, and data collection for reproductive health in Guatemala. They track how reproductive health policies are being put into practise, work to empower communities, and demand responsibility for information and services related to reproductive health. Local OSARs monitor activities associated with local priorities, engage in public education (e.g., promoting reproductive rights, reducing sexual and gender-based violence, etc.), advocacy and lobbying (e.g., pressuring the government for particular policies or legal frameworks, etc.), and collect data (e.g., tracking service quality or contraceptive stock-outs) that could provide supplementary information about family planning and reproductive health. | Supplementary |
|  | 3.7.2 Adolescent birth rate (aged 10–14 years; aged 15–19 years) per 1,000 women in that age group | **Could Contribute:** Saving Mothers Giving Lives (SMGL),  San Antonio Teen Pregnancy Collaborative (TX-US) | The SMGL initiative can be used for monitoring this indicator, as it has collected data on newborn and child services. As part of SMGL, about 4000 VHTs, one per village, were trained to provide preventative maternal and child health services and to report on the deaths of women of reproductive age. The San Antonio Teen Pregnancy Collaborative project (SATPPC) aims to reduce the Bexar County teen birth rate among females aged 15 to 19 by 15% by 2020. The SATPPC included a list of cross-sector organizations, including: public entities, community-based organizations, as well as faith-based and secular institutions. The SATPPC monitors the teen birth rates within three widely accepted age categories (10-14, 15-17, 15-19). The data resulting from SATPCC could provide supplementary information for monitoring this indicator. | **Could Contribute:**  Advancing Partners & Communities (APC) Uganda | <https://www.advancingpartners.org/sites/default/files/sites/default/files/resources/ugandafinalreport.pdf> | In addition to the projects highlighted in the 2020 sytematic review, Advancing Partners & Communities (APC) aimed to produce a national fertility hotspot map to identify regions and districts with the highest fertility rates and highest teenage pregnancy rates. Implementing and evaluating treatments that address the sociocultural causes of Uganda's high total fertility rate with a focus on low-parity mothers and youth aged 15 to 19 in high-risk areas was the goal. To effect changes on family planning, the project involved important gatekeepers and influencers at the community (village), subcounty, and district levels. Such projects are action-oriented, which would support the relevant target, but they could also provide useful information on adolescent birth rates and their locations. | Direct |
| 3.8 Achieve universal health coverage, including financial risk protection, access to quality essential health-care services and access to safe, effective, quality and affordable essential medicines and vaccines for all | 3.8.1 Coverage of essential health services | **Could Contribute:**  CBM on Quality of Care in Family Planning Services - India,  Engage TB | There are 14 tracer indicators determined for monitoring this indicator. They include some other SDG indicators such as 3.7.1 (Percentage of women of reproductive age (15−49 years) who are married or in a union who have their need for family planning satisfied with modern methods), 3.a.1 and 3.d.1, as well as some relevant ones such as tuberculosis (TB), HIV, malaria, hospital access, health workforce, etc. For 3.7.1, the CBM on Quality of Care in Family Planning Services - India project has already been identified whose data could be used. In addition, for the tuberculosis projects such as Engage-TB, which aims to screen for TB and TB-related morbidity (e.g., HIV counselling and testing, diabetes) including through home visits and referral for diagnosis of TB and related diseases, linking this with clinics, transport support and facilitation and accompaniment, the use of referral forms could be used for monitoring the tuberculosis tracer indicator as identified in 3.3.1 and 3.3.2. These are just a few examples. Given the broad scope of this indicator, there may be more citizen science projects to support the monitoring of this indicator if aimed at what this indicator is trying to measure. | **Could Contribute:**  Reproductive Health Observatories (Guatemala),  Ritshidze Project,  Community Treatment Observatory (CTO) and others... | see 3.3.1, 3.3.2, 3.3.3, 3.7.1, etc. | In addition to the projects highlighted in the 2020 systematic review, due to the broad scope of the indicator covering several other SDG indicators fully or partly (family planning, pregnancy and delivery care, child immunization, child treatment, tuberculosis, HIV/AIDS, malaria, water and sanitation, hypertension, diabetes, tobacco, hospital access, health workforce and health security), some of the projects highlighted in 3.3.1, 3.3.2, 3.3.3 and 3.7.1, among others, can support the monitoring of this indicator (see the justification for these indicators and other relevant ones as higlighted above). | Direct and Supplementary |
|  | 3.8.2 Proportion of population with large household expenditures on health as a share of total household expenditure or income | **No alignment** identified with any existing or past citizen science project. | N/A | **No alignment** identified with any existing or past citizen science project. | N/A | No alignment identified with any existing citizen science project. | N/A |
| 3.9 By 2030, substantially reduce the number of deaths and illnesses from hazardous chemicals and air, water and soil pollution and contamination | 3.9.1 Mortality rate attributed to household and ambient air pollution | **Could Contribute:** AirCasting, AirVisual, SmartCitizen, iSCAPE,  HackAir (but already finished) + CSIRO Energise, BioVill.Eu,  ISABEL | Given that both 11.6.2 and 7.1.2 have citizen science projects that could be used to support these indicators, it follows that they could also support this indicator. However, the data on air pollution is mostly used to model mortality and not to actually capture mortality. | **Could Contribute:**  IQAir,  CanAirIO | <https://www.iqair.com/air-quality-community>  <https://canair.io/> | This indicator is modeled by the WHO using “recorded measurements of particulate matter that is approximately 2.5 and 10 microns or less in diameter (abbreviated as PM2.5 and PM10, respectively) from official pollution monitoring stations around the world, incorporating around 3000 station inputs”.  In addition to the projects identified in the 2020 review, IQAir and CanAirIO and many other citizen science projects that aim to measure air quality can contribute to the monitoring of this indicator. | Direct |
|  | 3.9.2 Mortality rate attributed to unsafe water, unsafe sanitation and lack of hygiene (exposure to unsafe Water, Sanitation and Hygiene for All (WASH) services) | **No alignment** identified with any existing or past citizen science project. | N/A | **Could Contribute:**  Projects identified in 6.1.1 and 6.1.2,  Community education intervention to improve drinking water quality in rural communities in Puerto Rico | <https://www.ncbi.nlm.nih.gov/pmc/articles/PMC2876105/> | According to the metadata, this indicator is based on both the WASH service provision in a country, as well as the related health outcomes, and therefore provides important information on the actual disease caused by the risks measured in targets 6.1, 6.2 and 6.3. Metadata also highights that data rely on (a) statistics on WASH services (6.1, 6.2 and 6.3), and (b) data on deaths. Therefore, such projects identified as "could contribute" for indicators 6.1.1 and 6.1.2 can help provide supplementary information for this indicator.  Additionally, as part of a community education intervention to improve drinking water quality in rural communities in Puerto Rico, the communities that rely on communal water supplies received an educational intervention. The outcomes demonstrated that educational interventions targeting both specific individuals and the community as a whole in small communities with poor water quality are a means of empowering communities to manage their own drinking water quality, which is more focused on achieving the target than on its monitoring. | Supplementary |
|  | 3.9.3 Mortality rate attributed to unintentional poisoning | **No alignment** identified with any existing or past citizen science project. | No alignment identified with any existing citizen science project. There are citizen science projects (see rationale for indicator 3.5.1, e.g., OD Help, A-Chess, etc.) that focus on drug overdoses as they are the majority of unintentional poisoning deaths. They are, however, focusing on the impact in terms of reducing these incidences, not necessarily monitoring the mortality rate using citizen science. | **Could Contribute:**  Project Lazarus,  A-CHESS | <https://doi.org/10.1111/j.1526-4637.2011.01128.x>  <https://www.projectlazarus.org/home> | For unintentional poisioning, the indicator metadata refers to the International Classification of Diseases, Tenth Revision (ICD-10) codes X40, X43, X46-X48, X49, which includes exposure to drugs, among others.  Established in 2007, as a response to extremely high overdose mortality rates in Wilkes County, NC, Project Lazarus decreased Wilkes’ overdose mortality rate by devising and implementing the Project Lazarus Model, which is a public health model that involves communities to address the problem. As part of the project, the communities are empowered through, for instance, a Working Coalition made up of interested advocates, parents, youth, individuals in recovery, pain patients, etc. from the community. The overdose prevention program for the project consisted of five parts: community mobilisation and coalition building; monitoring and surveillance data; overdose prevention; community member usage of rescue medication to reverse overdoses; and evaluation of project components. The key initiatives include educating primary care doctors on how to manage chronic pain and safely prescribe opioids, mostly through the development of a toolkit and in-person workshops. Such programs do not only encourage action to address the issue of unintentional poisoning but also assist in monitoring poisoning.  Additionally, not necessarily for the mortality rate, but the project A-CHESS highlighted in 3.5.1 focuses on drug overdose, and can provide supplementary information for this indicator. | Supplementary |
| 3.a Strengthen the implementation of the World Health Organization Framework Convention on Tobacco Control in all countries, as appropriate | 3.a.1 Age-standardized prevalence of current tobacco use among persons aged 15 years and older | **No alignment** identified with any existing or past citizen science project. | N/A | **Could Contribute:**  A Coproduction Community-based Approach to Reducing Smoking Prevalence in a Local Community Setting | <https://www.hindawi.com/journals/jeph/2016/5386534/> | Professional researchers worked with community members who were interested in volunteering as "community researchers" in a project in North East England that aimed to create a community-led survey to understand smoking prevalence within the community, as well as examining attitudes towards smoking cessation, smoking in public places, and other topics. Following training, community representatives worked as volunteers to conduct the baseline survey. A variety of questions were posed to participants on their use of e-cigarettes, how much they smoked, where they purchased their cigarettes, and whether they were current smokers. Meeting needs and providing treatment are the foundation of the conventional approach to providing services to enhance health, with "experts" joining the project as outsiders to "fix" the community members. However, research has shown that by working with communities, it is possible to develop services that are meaningful to the community. Such community oriented projects are not only helpful in providing the data needed for the indicator at a community level in high granularity, but they can also support local level actions which can then be upscaled. | Direct |
| 3.b Support the research and development of vaccines and medicines for the communicable and non-communicable diseases that primarily affect developing countries, provide access to affordable essential medicines and vaccines, in accordance with the Doha Declaration on the TRIPS Agreement and Public Health, which affirms the right of developing countries to use to the full the provisions in the Agreement on Trade-Related Aspects of Intellectual Property Rights regarding flexibilities to protect public health, and, in particular, provide access to medicines for all | 3.b.1 Proportion of the target population covered by all vaccines included in their national programme | **Could Contribute:**  My Village My Home,  MAHEFA (MA-lagasy HE-althy FA-milies), MAHEFA Miaraka | The My Village My Home project offers a tool in the form of poster-sized material used by volunteers and health workers in a community to record the births and vaccination dates of every infant in a community. The introduction of the tool has allowed community leaders, volunteers, and health workers to monitor the vaccination status of every young child and has guided reminders and motivational visits in these communities. Therefore, My Village My Home could be used for monitoring this indicator. MAHEFA (MA-lagasy HE-althy FA-milies) is a community-based health project as outlined in 3.3.3. For example, community health volunteers in Analalava were tasked with a wide array of services that had previously been lacking, including raising childhood immunization rates. Therefore, MAHEFA could be used for monitoring this indicator as well. | **Could Contribute:**  Zoe Health Study | <https://www.afro.who.int/sites/default/files/2017-12/MCIA%20Brief_Role%20of%20Communities%20in%20Coverage%20and%20Demand.pdf>  <https://health-study.joinzoe.com/>  <https://doi.org/10.1016/S0140-6736(22)00327-0> | This indicator aims to measure the coverage of various vaccines. In addition to the highlighted projects in the 2020 systematic review, the Zoe Health Study uses a citizen science approach to tackle global health issues, including Covid-19. With over 4.7 million contributors from the UK, the project has published over 40 scientific papers in journals such as The Lancet, Nature Medicine, The Oncologist and Nature Food covering topics ranging from cancer and COVID risk, to the side-effects of COVID vaccines to vaccine effectiveness over time. As part of the project, participants are requested to download the Zoe Health Study app, and upon enrolment, they are asked to provide basic demographic and health information. Then, they provide daily updates on symptoms experienced, test results, vaccines administered, and if they were self-quarantining or seeking health care, including the level of intervention and related outcomes. Although Covid-19 is not among the diseases highlighted in the metadata of this indicator, similar approaches can help understand vaccine coverage for various diseases, with additional information that can help scientists to understand risks, vaccine effectiveness and side effects, among other issues and can complement official data related to the indicator. | Direct and Supplementary |
|  | 3.b.2 Total net official development assistance to medical research and basic health sectors | **No alignment** identified with any existing or past citizen science project. | N/A | **No alignment** identified with any existing or past citizen science project. | N/A | N/A | N/A |
|  | 3.b.3 Proportion of health facilities that have a core set of relevant essential medicines available and affordable on a sustainable basis | **No alignment** identified with any existing or past citizen science project. | N/A | **Could Contribute:**  Watch What Matters | <https://itpcglobal.org/wp-content/uploads/2020/10/ITPC-2020-They-Keep-Us-On-Our-Toes.pdf>  <https://itpcglobal.org/our-work/watch-what-matters/> | The indicator is based on the proportion of facilities, such as pharmacies, hospitals, clinics, primary care centers, public/private, etc., where core essential medicines are available for purchase and their prices are affordable. Citizen science and community-led approaches can help to uncover issues related to access to care and treatment and keep those in power accountable to the communities they serve.  For example, Watch What Matters is a project that gathers data on access and quality of HIV treatment globally. Acoording to the WHO Essential Medicines List, HIV medicine is an essential medicine (https://www.who.int/publications/i/item/WHO-MHP-HPS-EML-2021.02). Led by the International Treatment Preparedness Coalition (ITPC), the project intends to streamline and standardise treatment access data on HIV that are collected by communities in order to ensure that data are no longer gathered in a fragmented way. It is based on a methodology that gives communities the tools they need to regularly and systematically gather information on access barriers, analyse it for trends, inform advocacy work, and encourage accountability. So far, in South Asia, Eastern Europe and Central Asia, ITPC has monitored supply chain management issues and drug stock outs, and new community treatment observatories (CTOs) have been established in other parts of the world, such as in East Africa, West Africa, Central Africa and Latin America. This example shows that citizen science approaches have the potential not only to support the monitoring of the indicator, but also contribute to the action needed for the achievement of the relevant target. | Supplementary |
| 3.c Substantially increase health financing and the recruitment, development, training and retention of the health workforce in developing countries, especially in least developed countries and small island developing States | 3.c.1 Health worker density and distribution | **No alignment** identified with any existing or past citizen science project. | N/A | **No alignment** identified with any existing citizen science project. | N/A | N/A | N/A |
| 3.d Strengthen the capacity of all countries, in particular developing countries, for early warning, risk reduction and management of national and global health risks | 3.d.1 International Health Regulations (IHR) capacity and health emergency preparedness | **No alignment** identified with any existing or past citizen science project. | N/A | **No alignment** identified with any existing or past citizen science project. | N/A | N/A | N/A |
|  | 3.d.2 Percentage of bloodstream infections due to selected antimicrobial-resistant organisms | This indicator was included in the SDG Indicator Framework after the 2020 systematic review was completed. | New indicator/Not mapped in 2020. | **Could Contribute:**  Using Citizen-Generated Data to Address Antimicrobial Resistance in Kenya | <https://www.data4sdgs.org/resources/people-power-using-citizen-generated-data-address-antimicrobial-resistance-kenya> | The indicator metadata refers to National Antimicrobial Resistance (AMR) data collected through the national AMR surveillance system and reported to GLASS as the preferred data source. GLASS collects official national AMR data primarily through surveillance systems. However, more than half of the countries in Africa do not submit data on AMR to GLASS. AMR monitoring capacity in low- and middle-income countries (LMICs) varies for a variety of reasons, with poor laboratory capacity to support diagnostic microbiology being a prominent one. Complementary strategies are required to produce high-quality data on AMR as countries expand their monitoring data systems and new research on the disease is done. Citizen science/citizen-generated data approaches can play a crucial role in addressing these gaps, as well as understanding citizen knowledge levels, their perceptions and drivers of behaviour. Engaging citizens on AMR data activities can help close AMR data gaps, support citizens to take action on AMR, and spur data-driven action by political leaders. The Global Partnership for Sustainable Development Data collaborated with Africa’s Voices Foundation to generate and disseminate insights on AMR in Kenya through citizen participation. The citizen-generated data activities used were a combination of interactive radio shows in local languages and an SMS/mobile text messaging service, group discussions, and key informant interviews across three counties: Kiambu, Kilifi, and Bungoma. Over ten weeks, 38 interactive radio shows were hosted and 20,272 SMS from 5,313 individuals were generated. The project findings demonstrate the significant advantages of public involvement in AMR. Even in just 10 weeks, the project's understanding of AMR grew. Citizens genuinely wanted to learn more about AMR. This indicates how deliberate public education about AMR can close knowledge gaps and ultimately influence behaviour to combat AMR. By altering their habits regarding how they get, use, and misuse antimicrobials, citizens can be given the power to contribute to the fight against AMR. Such projects that connect citizen data to the AMR issue can help support the indicator as well as achieving the target. | Direct |
| **Goal 4. Ensure inclusive and equitable quality education and promote lifelong learning opportunities for all** | | | | | | | |
| 4.2 By 2030, ensure that all girls and boys have access to quality early childhood development, care and pre-primary education so that they are ready for primary education | 4.2.1 Proportion of children aged 24–59 months who are developmentally on track in health, learning and psychosocial well-being, by sex | **Could Contribute:**  CBM in Rural Jamaica on children growth | This indicator looks at the physical health, learning and social-emotional wellbeing of children under 5. For the physical health part of the indicator, there is a community growth monitoring activity, which is a volunteer program in rural Jamaica. As part of the program, primary school graduate volunteers were chosen by the community from the community to collect data on children's growth and to improve the nutritional status of children. | **Could Contribute:**  The Uganda Program for Human and Holistic Development (UPHOLD) | <https://www.sciencedirect.com/science/article/abs/pii/S0033350605800046>  <https://pdf.usaid.gov/pdf_docs/PA00SXW7.pdf> | In addition to the projects highlighted in the 2020 systematic review, a USAID funded program, the Uganda Program for Human and Holistic Development (UPHOLD), can be an example of how citizen science or community-based monitoring approaches can support the monitoring of child growth, which is one of the components of the indicator. As a catalyst for addressing issues with illness, poor feeding habits, or other childcare concerns at the community and household levels, such projects can assist communities in preventing malnutrition among children under the age of two. The initiative supported community-based growth promotion in a number of districts across its 34 districts of operation in Uganda. As part of the project, a total of 1,290 growth promoters were trained in 524 villages within 6 districts and they established monthly growth promotion sessions. | Supplementary |
| **Goal 5. Achieve gender equality and empower all women and girls** | | | | | | | |
| 5.2 Eliminate all forms of violence against all women and girls in the public and private spheres, including trafficking and sexual and other types of exploitation | 5.2.1 Proportion of ever-partnered women and girls aged 15 years and older subjected to physical, sexual or psychological violence by a current or former intimate partner in the previous 12 months, by form of violence and by age | **Could Contribute:** Safecity,  HarrasMap | Safecity (from India) allows reporting on sexual violence and HarrasMap allows incidents of sexual harassment or interventions to be reported; information on when someone has intervened to stop a sexual harassment incident or supported the harassed person can help to monitor the indicator. | **Already Contributing:**  Let's Talk  **Could Contribute:**  Digital Democracy/KOFAVIV Haiti 572, Clique 180 | <https://paris21.org/sites/default/files/inline-files/Gloria%20CGD%20Project%20on%20GVB_GDN_Ghana.pdf>  <https://www.isglobal.org/documents/10179/25254/Innovative+Community-Based++Approaches+to+Addressing+Access++to+Sexual+Violence+Services/da7a7506-1d09-4117-8426-3250e77562e5>  <https://www.unwomen.org/sites/default/files/Headquarters/Attachments/Sections/Library/Publications/2015/UN%20Women%20_Step%20It%20Up%20Brochure_Rd8_snglpgs-web.pdf> | In addition to the projects highlighted in the 2020 systematic review, the Ghana Statistical Service (GSS) has used citizen-generated data/citizen science approaches to collect data related to this indicator (5.2.1), as well as indicators 5.2.2, 11.7.2 and 16.2.3. As part of the project, pilot work was conducted in three districts across all 3 ecological zones of Ghana (Ho, Techiman, and Central Gonja) and the project is planned to be rolled-out nationwide. In the project, through a design-thinking process, an app and an IVR service, called Let's Talk, was developed, where users anonymously recorded information related to gender based violence. Results were compared to the data collected through traditional means and showed that through such approaches, more data can be collected, as women may not officially report on the violence they face. One of the advantages of citizen science approaches identified by the GSS through the project was that people are willing to report on behalf of others’ experiences of gender based violence, which is not available through official surveys and official domestic violence reports in Ghana.  Additionally, the Digital Democracy/ KOFAVIV Haiti 572 project is a rape crisis hotline providing information for survivors. Designed for mobile phones, calls are answered 24 hours a day and free from the main Haiti service providers. The project collects sexual and gender based violence data and allows local women to track, analyze, map and share data on incidents of violence.  Clique 180 by UN Women is a free Smartphone application, which includes nationwide services and information indicating which local, state-level or federal public services, non-governmental or academic resources are located closest to the user, their hours of operation and how to get there. It is designed for addressing sexual and gender based violence. | Direct |
|  | 5.2.2 Proportion of women and girls aged 15 years and older subjected to sexual violence by persons other than an intimate partner in the previous 12 months, by age and place of occurrence | **Could Contribute:** Safecity,  HarrasMap | Same as 5.2.1 | **Already Contributing:**  Let's Talk  **Could Contribute:**  Digital Democracy/KOFAVIV Haiti 572, Clique 180 | See above 5.2.1 links. | In addition to the projects highlighted in the 2020 systematic review, projects mapped in 5.2.1 can also support this indicator. Please see the 5.2.1 justification for further information. | Direct |
| 5.3 Eliminate all harmful practices, such as child, early and forced marriage and female genital mutilation | 5.3.1 Proportion of women aged 20–24 years who were married or in a union before age 15 and before age 18 | **No alignment** identified with any existing or past citizen science project. | N/A | **Could Contribute:**  ActionAid’s Ending Child Marriage Program | <https://www.unicef.org/media/109586/file/Technical-note-monitor-child-marriage-programmes-COVID-19-2021.pdf> | ActionAid’s Ending Child Marriage programme used its network of 52 district coordinators in four states across India. To establish task teams to stop child marriages, the coordinators collaborated closely with the local authorities. Additionally, they gave community members training and increased understanding of child marriage in the neighbourhood. Data from the program reveals that, of the 205 child weddings reported during the Covid shutdown in West Bengal, 195 could have been avoided. Working with the support of UNICEF, the ActionAid Association (AAA) launched the project “Strengthen institutional capacity of key district government stakeholders to enhance adolescent empowerment and to prevent child marriage” in 2019, in the states of Bihar, Odisha, Rajasthan and West Bengal. Such programs can support both monitoring efforts in communities and also help to achieve the relevant target. | Direct |
|  | 5.3.2 Proportion of girls and women aged 15–49 years who have undergone female genital mutilation/cutting, by age | **No alignment** identified with any existing or past citizen science project. | N/A | **Could Contribute:**  Technology - and community - empowered programming to eliminate FGM in Kuria Kenya,  USAID's Healthy Unions Project | <https://www.icrw.org/wp-content/uploads/2021/02/LastMile4D_ICRW_Technology-and-community-empowered-programming-to-eliminate-FGM_02.2021.pdf>    <https://www.lastmile4d.org/index.html?r=V25>  <https://www.actionaidindia.org/sponsor-a-child-new/> | Technology - and community - empowered programming to eliminate female genital mutilation (FGM) in Kuria Kenya was conducted as a pilot program by LastMile4D. The programme included three main elements: 1) education to encourage target communities to end FGM, 2) periodic, in-person monitoring of a group of schoolgirls at risk of FGM by trained field workers, and 3) crisis intervention, including a hotline, a point of contact for law enforcement, and alternative housing for girls who were about to be cut. Staff from LastMile4D organised multiple educational seminars for community people, including parents, boys and girls, heads of schools, tribal and religious leaders, law enforcement, and members of the public. The meetings aimed to improve communication, inform attendees about FGM, and enable the community to develop a strategy to end FGM. The LastMile4D team used its patented VPack Technology, a secure online platform, to train field employees on data collection methods. VPack comes with a laptop, a transportable solar-powered backpack, and software that enables users to instantly gather and transmit information. During COVID-19, for example, this feature allowed field personnel to monitor girls in a variety of locations (such as at home or school). 1,090 schoolgirls enrolled in ten schools in Kuria, aged 9 to 17, were monitored by field workers. Using the same basic forms, program monitoring data were gathered three times in 2020, with the last two time points adding new questions about the effects of COVID-19. The effectiveness of combining technology, community-focused initiatives, ongoing outreach, and monitoring and evaluation to end FGM was proved by this pilot program. The team at LastMile4D discovered that timely intervention was made possible by effective data collection utilizing VPack and ongoing interaction with girls at risk of FGM. The majority of girls who would have otherwise been cut were probably able to continue in school for an additional year because FGM exposure was postponed by providing shelter to girls during the season when the risk of FGM is at its maximum. The findings demonstrated the importance of implementing community based FGM programmes with real-time monitoring for the benefit of young girls.  Additionally, USAID's Healthy Unions Project can also support this indicator. USAID’s Healthy Unions project implemented by CARE Ethiopia addressed the interlinked harmful traditional practices of bride abduction, bride price, and early marriage through conversations with the community and raising awareness on the harmful effects of such practices. The program, which was run by CARE Ethiopia and involved local NGOs, community-based organisations, local government officials, and schools, touched 84,000 individuals. In order to help prevent bride pricing, bride abduction, early marriage, and other detrimental practises, the project developed 78 community-level committees made up of volunteers. It also helped girls who got married young and had difficult pregnancies by locating and offering medical assistance. 75 cases of Female Genital Mutilation/Cutting (FGM/C) were brought to court and resulted in convictions thanks to the project's network of volunteers and cooperation with local authorities. Furthermore, because of community mobilization and awareness raising efforts, the project estimates that 75% of the FGM/C practitioners in the project area abandoned the practice. To advocate for the human and legal rights of women and girls, the project also provided paralegal training for 650 community volunteers. Such projects that are conducted mainly for action rather than monitoring, can still support the monitoring efforts related to the indicator and target at a local/community level, while at the same time contributing to the target. | Direct and Supplementary |
| 5.6 Ensure universal access to sexual and reproductive health and reproductive rights as agreed in accordance with the Programme of Action of the International Conference on Population and Development and the Beijing Platform for Action and the outcome documents of their review conferences | 5.6.1 Proportion of women aged 15–49 years who make their own informed decisions regarding sexual relations, contraceptive use and reproductive health care | **Could Contribute:** MAHEFA,  MAHEFA Miaraka, Community-based Monitoring (CBM) on Quality of Care in Family Planning Services India | Once geared towards the requirements of this indicator, projects identified and mapped as "could be used" for indicator 3.7.1 could support monitoring this indicator as well. Community-based Monitoring (CBM) on Quality of Care in Family Planning Services India aimed to identify quality of care in family planning. Data collected throughout the community scorecard process, before and after, provided information on women of reproductive age who have their need for family planning satisfied with modern methods (as the indicator requires). MAHEFA (MA-lagasy HE-althy FA-milies) could also provide supplementary information to this indicator, because one of it concerns was providing family planning services. The entire process was led by women selected from the community in their villages. These women were given orientation on the objectives of the process and provided training in the use of the tools and the process of administering them so the people that organized the process were from the community and they did it with community members. The data providers were not only volunteers but also the data collectors. | **Already Contributing:**  Women and Health in Uruguay (MYSU)  **Could Contribute:**  Reproductive Health Observatories (Guatemala); | <https://www.healthpolicyproject.com/pubs/449_PSocialAccountabilityReportFINALEC.pdf>  <https://osarguatemala.org/embarazos-y-registro-de-nacimientos-2021/>  <https://sustainabledevelopment.un.org/content/documents/15781Uruguay2.pdf>    <https://paris21.org/sites/default/files/2021-02/CGD_FINAL_reduced.pdf> | In addition to the projects highlighted in the 2020 systematic review, the National Statistics Institute of Uruguay (Instituto Nacional de Estadística) used gender data from an NGO called Women and Health in Uruguay (MYSU) to report on some indicators under SDG 3 and SDG 5 in their 2017 Voluntary National Review (VNR) 2017 (VNR Uruguay, 2017- see the link in the previous column) including this particular indicator. The VNR stressed that data from MYSU, although not official, provide valuable insights on sexual and reproductive health that are relevant for the country. MYSU gathers data through surveys on sexual and reproductive health in order to track public policies and highlight the difficulties in providing public services related to sexual and reproductive health in Uruguay both for women and men.  Reproductive Health Observatories (Guatemala), as identified in 3.7.1, can contribute to the monitoring of this indicator. In Guatemala, OSARs (Observatorios en Salud Reproductiva, or Reproductive Health Observatories), independent civil-society-led bodies, were established at both the national and local levels through a memorandum of understanding with the government and supported by donor funding. They emphasize accountability, monitoring, and data collection for reproductive health in Guatemala. They track how reproductive health policies are being put into practise, work to empower communities, and demand responsibility for information and services related to reproductive health. Local OSARs monitor activities related to local priorities, and they engage in public education (e.g., promoting reproductive rights, reducing sexual and gender-based violence); advocacy and lobbying (e.g., exerting pressure on government for specific policies or legal frameworks); and data collection (e.g., monitoring the quality of services or contraceptive stock-outs), which could support supplementary information related to family planning and reproductive health. | Direct |
|  | 5.6.2 Number of countries with laws and regulations that guarantee full and equal access to women and men aged 15 years and older to sexual and reproductive health care, information and education | **No alignment** identified with any existing or past citizen science project**.** | N/A | **No alignment** identified with any existing or past citizen science project**.** | N/A | N/A | N/A |
| **Goal 6. Ensure availability and sustainable management of water and sanitation for all** | | | | | | | |
| 6.1 By 2030, achieve universal and equitable access to safe and affordable drinking water for all | 6.1.1 Proportion of population using safely managed drinking water services | **Could Contribute:** Freshness of Water,  Aqua | Citizen science projects such as Aqua and Freshness of Water “can be used” to monitor this indicator. The indicator is about basic drinking water sources located at the point of collection within the dwelling, yard or plot. The Aqua (Spain) initiative is aimed at controlling the quality of drinking water. Thousands of Spanish participants, particularly students, created a map with the measurements they made on water quality (chlorine, pH, flavor, smell). The Freshness of Water (NL) initiative, on the microbiological stability of drinking water, enabled citizens to analyze samples from their own kitchen tap and test the water quality using test strips. | **Could Contribute:**  Using Citizen Science Approach to monitor water, sanitation and hygiene Related Risks in Karonga Town of Malawi,  Sanitary Inspections (SI) in Malawi,  Crowd the Tap | <https://academicjournals.org/journal/AJEST/article-full-text-pdf/99368D264363.pdf>  <https://www.mdpi.com/2079-9276/9/12/142/htm>  <https://crowdthetap.org/about/> | In addition to the projects highlighted in the 2020 systematic review, in a project implemented in Karonga Town in 2017, Malawi, a citizen science approach was used to integrate communities into scientific research on water quality and WASH related risk monitoring. Analyses of the water samples' biological, physical, and chemical characteristics were conducted. The water samples were analysed for biological, physical and chemical parameters. The findings revealed that Escherichia coli contamination levels in the majority of water samples from shallow wells, rivers and streams, lakes, and boreholes were significantly higher than those allowed by the Malawi Bureau of Standards for drinking water. The outcomes also showed that untreated water is not fit for direct human consumption.  In another citizen science project impelemented in Malawi on Sanitary Inspections (SI), a mixed methods approach of quantitative on-site SI data collection and remote SI data collection via photographic images was used, along with qualitative data collected by citizen scientists and a panel of experts in the field of SI. Results showed that there is potential for citizen science supported SI, with remote expert verification of the results using photographic images.  Crowd The Tap is another citizen science project example that could support this indicator. As part of the project that focuses on identifying and addressing lead contamination in household drinking water, scientists use the information about the age of a house and the pipes in the house to determine the risk of lead contamination. Anyone found to be at risk for lead contamination receives free lead-in-water testing of their household tap water while funding is available.  Such citizen science projects and many others from many different parts of the world could support 6.1.1 monitoring. | Direct |
| 6.2 By 2030, achieve access to adequate and equitable sanitation and hygiene for all and end open defecation, paying special attention to the needs of women and girls and those in vulnerable situations | 6.2.1 Proportion of population using (a) safely managed sanitation services and (b) a hand-washing facility with soap and water | **Could Contribute:** Action Research for Learning - Bangladesh | Action Research for Learning can be mapped as could contribute as the project includes monitoring activities through a CBM system, where women volunteers in five selected villages in Bangladesh visited 60 neighbours each month to ask where they collect drinking water, looked at the condition of the toilet, checked that all household members are using it and asked about handwashing facilities. In addition, they conducted weekly sessions on good hygiene behaviour in 20 schools in the Satkhira district. To track progress of this activity, student volunteers interviewed ten boys and girls in five selected schools each month to find out what they think about the toilets and the state of hygiene in their school. The community and school volunteers then shared their findings so that an up-to-date picture of access to safe water, clean toilets and hygiene practices in villages and schools is generated. | **Could Contribute:**  Sanitary Inspections (SI) in Malawi  Mukuru Special Planning Area,  Integrated Development Plan in Nairobi | <https://www.mdpi.com/2079-9276/9/12/142/htm>  <https://doi.org/10.3390/urbansci6020036> | In addition to the projects highlighted in the 2020 systematic review, the Sanitary Inspections (SI) in Malawi project, as presented in 6.1.1 above, can also contribute to this indicator and target. See 6.1.1 above for further information on the project.  In another project, a citizen-science, climate justice planning process in the Mukuru informal settlement of Nairobi, Kenya was implemented. The initiative produced evidence to support an integrated, climate justice plan known as the Mukuru Special Planning Area, Integrated Development Plan by co-creating data-gathering procedures with citizens. The citizen science methods revealed that about 37% of inhabitants lacked regular access to safe and affordable drinking water and that only 1% of residents had a private in-home toilet. Additionally, 39% of homes reported fair or poor health, 42% of households experienced regular flooding, and 40% of households reported having a child that was stunted. Thousands of locals took part in the project and worked together to co-design improvement and climate change adaptation methods, such as flood mitigation, formalising roads and pathways with drainage, and a water and sanitation infrastructure plan for everyone. Citizen scientists then used these data and moved this evidence into actions to protect human health and drafted a climate justice strategy. | Direct |
| 6.3 By 2030, improve water quality by reducing pollution, eliminating dumping and minimizing release of hazardous chemicals and materials, halving the proportion of untreated wastewater and substantially increasing recycling and safe reuse globally | 6.3.1 Proportion of domestic and industrial wastewater flows safely treated | **Could Contribute:** Action Research for Learning - Bangladesh | This indicator is about the proportion of wastewater generated by households and by economic activities that is safely treated. The household portion of wastewater is the same indicator as 6.2.1 so data on the treatment of domestic wastewater will come from 6.2.1. Hence projects such as Action Research for Learning Bangladesh is mapped as “could be used” here. For more information on this project and how it is linked to the monitoring of this indicator, please see indicator 6.2.1. | **Could Contribute:**  Sanitary Inspections (SI) in Malawi  Mukuru Special Planning Area, Integrated Development Plan in Nairobi | <https://www.mdpi.com/2079-9276/9/12/142/htm>  <https://doi.org/10.3390/urbansci6020036> | In addition to the projects highlighted in the 2020 systematic review, the Action Research for Learning - Bangladesh, the Sanitary Inspections (SI) in Malawi and the Mukuru Special Planning Area and Integrated Development Plan in Nairobi projects presented in 6.2.1 above or other similar approaches can also provide supplementary information for this indicator. This is because the "proportion of wastewater generated by households" portion of wastewater is the same indicator as 6.2.1 so data on the treatment of domestic wastewater will come from 6.2.1. | Direct |
| 6.a By 2030, expand international cooperation and capacity-building support to developing countries in water- and sanitation-related activities and programmes, including water harvesting, desalination, water efficiency, wastewater treatment, recycling and reuse technologies | 6.a.1 Amount of water- and sanitation-related official development assistance that is part of a government-coordinated spending plan | **No alignment** identified with any existing or past citizen science project**.** | N/A | **No alignment** identified with any existing or past citizen science project**.** | N/A | N/A | N/A |
| 6.b Support and strengthen the participation of local communities in improving water and sanitation management | 6.b.1 Proportion of local administrative units with established and operational policies and procedures for participation of local communities in water and sanitation management | **No alignment** identified with any existing or past citizen science project**.** | N/A | **No alignment** identified with any existing or past citizen science project**.** | N/A | N/A | N/A |
| **Goal 7. Ensure access to affordable, reliable, sustainable and modern energy for all** | | | | | | | |
| 7.1 By 2030, ensure universal access to affordable, reliable and modern energy services | 7.1.2 Proportion of population with primary reliance on clean fuels and technology | **Could Contribute:** CSIRO Energise | The CSIRO Energise app referred to in 7.1.1. asks citizens about solar and wood. Please see the rationale for indicator 7.1.1 for more information. | **Could Contribute:**  CSIRO Energize,  URwatair | <https://www.csiro.au/en/Showcase/CSIRO-Energise>  <https://www.researchgate.net/publication/355889929_A_Citizen_Science_Approach_for_Indoor_Air_Quality_in_Thessaloniki_Greece> | Because the indicator has a component related to "access to clean fuels and technology" (also covered as part of indicator 1.4.21, CSIRO Energise can also contribute to the helath related aspect of this indicator. CSIRO Energise, implemented in Australia, has helped scientists to better understand how households across the country use, generate and interact with energy. The data provided by citizen scientists has been crucial in order to conduct research towards a more secure, reliable and sustainable energy future for Australia. The app produced data on household energy use including on appliance usage and energy bills, through to the emergence of new technologies.  Additionally, the URwatair project for Indoor Air Quality in Thessaloniki can be a good example for supporting this indicator. The metadata of the indicator highlights the importance of measuring indoor air quality. As part of the URwatair project, scientists explored the causes of indoor pollution to provide solutions regarding reduction of risk to indoor health concerns. In the project, citizens utilized low-cost sensors to monitor the quality of the air inside their buildings, living and cooking areas. Such projects can help contexualize the indicator and help raise awareness among citizens related to their daily practices. | Supplementary |
| **Goal 8. Promote sustained, inclusive and sustainable economic growth, full and productive employment and decent work for all** | | | | | | | |
| **Goal 9. Build resilient infrastructure, promote inclusive and sustainable industrialization and foster innovation** | | | | | | | |
| **Goal 10. Reduce inequality within and among countries** | | | | | | | |
| **Goal 11. Make cities and human settlements inclusive, safe, resilient and sustainable** | | | | | | | |
| 11.6 By 2030, reduce the adverse per capita environmental impact of cities, including by paying special attention to air quality and municipal and other waste management | 11.6.2 Annual mean levels of fine particulate matter (e.g. PM2.5 and PM10) in cities (population weighted) | **Could Contribute:** AirCasting, AirVisual, SmartCitizen, iSCAPE,  HackAir (there are other citizen science projects measuring air pollution but not PM2.5 or PM10) | These projects measure PM2.5 so could be directly incorporated into the WHO model, which uses PM2.5 measurements from authoritative stations. This could result in a much denser distribution in many cities than would be supplied to WHO. The only major issue would be about the quality of these sensors. | **Could Contribute:** IQAir,  CanAirIO | <https://www.iqair.com/air-quality-community>    <https://canair.io/> | This indicator is related to 3.9.1 presented above, and as highlighted in the 2020 systematic review, the projects mentioned and many other similar ones measure PM2.5 so could be incorporated into the WHO model, which uses PM2.5 measurements from authoritative stations. This could result in a much denser distribution in many cities than would be supplied to WHO. However, the quality of these sensors should be investigated. | Direct |
| **Goal 12. Ensure sustainable consumption and production patterns** | | | | | | | |
| **Goal 13. Take urgent action to combat climate change and its impacts** | | | | | | | |
| **Goal 14. Conserve and sustainably use the oceans, seas and marine resources for sustainable development** | | | | | | | |
| **Goal 15. Protect, restore and promote sustainable use of terrestrial ecosystems, sustainably manage forests, combat desertification, and halt and reverse land degradation and halt biodiversity loss.** | | | | | | | |
| **Goal 16. Promote peaceful and inclusive societies for sustainable development, provide access to justice for all and build effective, accountable and inclusive institutions at all levels** | | | | | | | |
| 16.1 Significantly reduce all forms of violence and related death rates everywhere | 16.1.1 Number of victims of intentional homicide per 100,000 population, by sex and age | **Could Contribute:**  Gun Violence Database | The Gun Violence Database project could be used for the monitoring of this indicator through providing supplementary information. This project combines machine learning and crowdsourcing techniques to produce a national registry of shooting incidents in the USA. The project collects information on both deaths and non-fatal shootings. Presumably, in the USA, almost all fatal shootings would be recorded in official statistics. However, this could be a valuable approach in other countries where national data collection systems are weak/non-existent. One consideration is that this project only measures firearm violence so it would be less useful for measuring this indicator in countries with low rates of firearm homicides (Burrows, 2019). However, it could be extended to include other types of violence to complement the indicator. | **Could Contribute:**  Gun Violence Database | <https://medium.com/penn-engineering/crowdsourcing-gun-violence-research-857a1cd87450> | As highlighted in the 2020 sytematic review, projects such as the Gun Violence Database could provide supplementary information for this indicator, even though it covers gun-based violence and not necessarily all intentional homicides. The project combines machine learning and crowdsourcing techniques to produce a national registry of shooting incidents in the USA. The project collects information on both deaths and non-fatal shootings. Presumably, in the USA, almost all fatal shootings would be recorded in official statistics. However, this could be a valuable approach where national data collection systems are weak/non-existent. One consideration is that this project only measures firearm violence so it would be less useful for measuring this indicator in countries with low rates of firearm homicides (Burrows, 2019). However, it could be extended to include other types of violence to complement the indicator. The 2022 review did not reveal any additional projects, although there may be others. | Supplementary |
|  | 16.1.3 Proportion of population subjected to (a) physical violence, (b) psychological violence and (c) sexual violence in the previous 12 months | **Could Contribute:** Safecity,  Gun Violence Database,  Ushaidi's crowdsourcing platform, HarrasMap | Initiatives such as Safecity, Gun Violence Database, Ushaidi's Crowdsourcing Platform and HarrasMap could provide complementary data to inform this indicator. Safecity (from India) reports on sexual violence while the Gun Violence Database combines machine learning and crowdsourcing techniques to produce a national registry of shooting incidents. Ushaidi's crowdsourcing platform allows violence reports to be submitted and the events to be mapped. HarrasMap allows incidents of sexual harassment or interventions to be reported. | **Already Contributing:**  Let's Talk  **Could Contribute:**  Digital Democracy/KOFAVIV Haiti 572, Clique 180 | See the links in 5.2.1 above. | In addition to the projects highlighted in the 2020 systematic review, the projects covered in 5.2.1 can also support this indicator. Please see the 5.2.1 justification for further information. | Direct |
| 16.2 End abuse, exploitation, trafficking and all forms of violence against and torture of children | 16.2.1 Proportion of children aged 1–17 years who experienced any physical punishment and/or psychological aggression by caregivers in the past month | **No alignment** identified with any existing or past citizen science project. | N/A | **Could Contribute:**  Reducing violence against children: Lessons from a community-based approach in post-conflict Uganda | <https://bettercarenetwork.org/sites/default/files/2019-09/Northern-Uganda-brief-FINAL.pdf> | Reducing violence against children: Lessons from a community-based approach in post-conflict Uganda showed that the intervention had a positive impact on caregivers’ knowledge of child abuse and a reduction in the use of physical punishment. Caregivers who showed a high level of understanding about child abuse increased by 9.2%. Through the development of a structure of shared responsibility and mutual support, the intervention proved helpful for child safety within communal life. In order to stop violence against children, this community-based strategy for protecting children placed a priority on building community capacity and emphasising consensus and cooperation. The initiative provided parents, carers, and community members with the opportunity to learn about and discuss the effects of violence on children's development as well as to come up with local solutions. The program offered direction, oversight, and support through the subsequent three phases: (i) Community-driven analysis of the type and frequency of violence, abuse, neglect and exploitation of children in the targeted communities, (ii) Dialogue sessions with community members that focus on building knowledge and (iii) changing attitudes towards child abuse. Although not intended for monitoring, such approaches can support action at a local/community level but they also have the potential to gather relevant information at the community level. | Supplementary |
| **Goal 17. Strengthen the means of implementation and revitalize the Global Partnership for Sustainable Development** | | | | | | | |
| **WHO Triple Billion Targets that are not SDG indicators** | | | | | | | |
|  | Health emergencies: Vaccine coverage for epidemic prone diseases | Triple Billion indicators were not part of the 2020 systematic review. | N/A | **Could Contribute:**  My Village My Home,  Covid-19 Community engagement and GIS in Michigan | <https://www.mcsprogram.org/resource/community-monitoring-of-individual-childrens-vaccinations/>  <https://doi.org/10.3390/tropicalmed7080177> | USAID's My Village My Home tool was intended to create a social expectation that families will keep their children up-to-date on vaccinations. The tool was intended to educate and inspire parents, community leaders, volunteers, and professional health workers to vaccinate infants more frequently and earlier. The child's name, birthdate, and dates of each vaccine were among the information gathered using the tool. Most often, a community census was carried out by local volunteers to gather the names and dates of birth of all infants. They then added these data to the tool. Between 2009 and 2019, the instrument was used in 6 different nations, including India, Timor-Leste, Malawi, Zimbabwe, Nigeria, and Tanzania. The project facilitated My Village My Home and other community-based monitoring approaches of infant immunization status in six countries, with generally positive results related to coverage, timeliness, and more accurate target populations, among others. Even though the project has covered child immunization only and not all age groups, it shows that citizen science/community-based approaches has/have the potential to complement official data with more granularity at a local/community level.  In another project related to Covid-19, community-engagement, and geographic information systems (GIS) strategies were implemented in order to increase equitable access to COVID-19 vaccination by decreasing the structural barriers to COVID-19 vaccine uptake in the US, with a particular focus on Asian Americans living in West and Southeast Michigan with low English proficiency. In order to reach underserved individuals in their communities, the project team established community-led mobile and pop-up COVID-19 vaccination clinics. They also collaborated with commercial pharmacies and scheduled appointments for community-based organisations, used GIS to establish COVID-19 vaccination sites close to communities with the greatest need, and deployed messengers to deliver linguistically and culturally appropriate COVID-19 vaccine messages, which increased vaccine confidence among the target population. This strategy improved availability to COVID-19 vaccination. The findings demonstrated that community-driven, cross-sector partnerships can improve efforts and make the COVID-19 vaccine more accessible to populations that are difficult to reach. This and other activities of a similar nature can aid in impact and monitoring. | Direct |
|  | Health emergencies: Proportion of vulnerable people in fragile settings provided with essential health services (%) | Triple Billion indicators were not part of the 2020 systematic review. | N/A | **Could Contribute:**  Know Your City,  Covid-19 Community engagement and GIS in Michigan,  WHO Snakebite Information and Data Platform | <https://cdn.who.int/media/docs/default-source/blue-print/who-covid-19-social-science-in-outbreak-report_15.08.21.pdf?sfvrsn=ddbb00b3_9&download=true>  <https://doi.org/10.3390/tropicalmed7080177>  <https://www.who.int/teams/control-of-neglected-tropical-diseases/snakebite-envenoming/snakebite-information-and-data-platform>  <https://www.who.int/publications/i/item/9789241515641> | The Know Your City (KYC) is an initiative by Slum Dwellers International (SDI) and United Cities and Local Governments (UCLG) supported by the Cities Alliance. The project helps Kenyan communities collect data, use it to enhance their neighbourhoods and settlements, and interact with local authorities to engage in more inclusive planning. Communities across Africa and Asia gathered geospatial and other information about urban informal settlements, including physical information about amenities like water and sanitation points and health facilities, population density, and boundary maps.  Additionally, in Kenya, SDIKenya and Accountability and Responsiveness in Informal Settlements for Equity (ARISE) collected data on the number and location of community health volunteers, which is combined with qualitative data on challenges, including training, resources and remuneration. All the research findings are validated by communities. The project has assisted communities that reside in unofficial settlements in getting those places listed on official city maps. These communities are now taken into account when planning for and making investments in the improvement of urban informal settlements as a result of this recognition by local authorities. The project has supplied city data on over 7,000 slums and informal settlements in 478 cities across 32 nations. Research findings in Kenya include settlement profiling information that assisted the country's COVID-19 National Task Force in identifying gaps and potential readiness actions as well as providing the Ministry of Health with information on the number of informal settlements in Nairobi during the COVID-19 crisis. The data produced as part of the project were also used by support organisations to plan interventions for handwashing, including handwashing stations, design of sensitisation campaigns for COVID-19, and supporting over 5,000 vulnerable families in the communities. Such iniaitives can provide data about vulnerable populations and their locations, and link them to the essential health services, especially in health emergencies, such as COVID-19.  Covid-19 Community engagement and GIS in Michigan that was presented as a project that could support the Triple Billion indicator "Health emergencies: Vaccine coverage for epidemic prone diseases" (see the above-mentioned indicator for details), can also support this indicator and action for the relevant target due to its particular focus on underserved communities.  Furthermore, the WHO Snakebite Information and Data Platform as presented in indicator 1.4.1 and others can also contribute to this indicator. Snakebites are particularly a problem for those with low socioeconomic status or poverty according to the WHO report (see the relevant link in the previous column). | Direct and Supplementary |
|  | WHA66.10 Prevalence of raised blood pressure in adults aged ≥18 | Triple Billion indicators were not part of the 2020 systematic review. | N/A | **Could Contribute:**  Blood Pressure Self-reporting Program Iran | <https://www.mdpi.com/1660-4601/18/18/9666#B68-ijerph-18-09666> | The Blood Pressure Self-reporting Program of “Iran National Mobilization for Controlling of Hypertension” initiative was implemented by the Iranian government in 2019 and attracted around 500,000 volunteers. The project is an example of how citizen science aproaches can support the monitoring of this indicator. | Direct |
|  | WHA66.10 Best practice policy implemented for industrially produced trans fatty acids (TFA) (Y/N) | Triple Billion indicators were not part of the 2020 systematic review. | N/A | **No alignment** identified with any existing or past citizen science project**.** | N/A | N/A | N/A |
|  | WHA66.10 Prevalence of obesity among children and adolescents (aged 5–19) (%) Prevalence of obesity among adults aged ≥18 | Triple Billion indicators were not part of the 2020 systematic review. | N/A | **Could Contribute:**  BigO,  EPODE,  Zoe's Big Diet Study | <https://bigoprogram.eu/big-data-against-childhood-obesity/>  <https://mhealth.jmir.org/2021/7/e26290>  <https://ieeexplore.ieee.org/document/9175361>  <https://www.eufic.org/en/healthy-living/article/preventing-childhood-obesity-through-community-based-initiatives>  <https://pubmed.ncbi.nlm.nih.gov/22106871/>  <https://health-study.joinzoe.com/blog/covid-big-diet-study> | For the projects BigO and EPODE identified as "could contribute" here, see the justification in 2.2.2 as to how these projects can contribute to this indicator.  Additionally, Zoe's Big Diet Study in the UK consisted of an in-depth survey looking at paricipants' own personal recent diet and food consumption habits to look at their gut health and regular diet patterns, as well as COVID data, to better understand how they link to the development of diseases like cancer. Even though the study was not necessarily designed to understand the prevalence of obesity, it collected data on personal health and eating habits from participants, which can provide useful information related to this indicator. The study aims to better understand the connection between cancer and diet. | Supplementary |
|  | WHA68.3 Number of cases of poliomyelitis caused by wild poliovirus | Triple Billion indicators were not part of the 2020 systematic review. | N/A | **Could Contribute:**  AVADAR – Auto Visual AFP Detection and Reporting,  Core Group Polio Partnership (CGPP) - Nigeria and in other countries such as Pakistan, Afghanistan, and others | <https://www.africakicksoutwildpolio.com/the-top-five-tech-solutions-that-helped-eradicate-wild-poliovirus-in-the-african-region/>  <https://bmcpublichealth.biomedcentral.com/articles/10.1186/s12889-018-6187-x>  <https://www.ncbi.nlm.nih.gov/pmc/articles/PMC6776102/> | Auto-Visual AFP Detection and Reporting (AVADAR) is a project and an SMS based technology, where some 10,000 trained community members, e.g., traditional healers, village leaders, etc., have been trained to use it. Using AVADAR on their mobile phones, community members report incidents of polio-like symptoms to the health ministries and WHO. Today, among other things, AVADAR has been used to identify and report cases of yellow fever, cerebrospinal meningitis, and Lassa fever. The technologies and extensive AVADAR community network have also been used during the COVID-19 epidemic. Although the project's emphasis is on polio prevention rather than counting the number of cases of poliomyelitis, it can nonetheless support both monitoring and action to reach this target.  In CGPP Nigeria, utilising "community structures" for monitoring and communication has improved immunisation acceptance in some of Nigeria's most challenging and high-risk polio locations. The volunteer community mobilizers (VCMs) implemented innovative strategies to ensure high vaccination coverage, such as community dialogues, community health camps, and tracking of noncompliant families, missed children, and dropouts. The VCMs in Nigeria’s polio initiative was an important contribution to reductions in the number of households rejecting polio immunization, the proportion of families with missed children, the proportion of families that were non-compliant, and the number of polio cases. The program was more focused on action rather than monitoring of existing cases, but such initiatives could provide an important contribution to polio eradication. | Supplementary |
|  | WHA68.7 Patterns of antibiotic consumption at national level | Triple Billion indicators were not part of the 2020 systematic review. | N/A | **Could Contribute:**  Using Citizen-Generated Data to Address Antimicrobial Resistance in Kenya,  The Antibiotic Smart Community Project | <https://www.data4sdgs.org/resources/people-power-using-citizen-generated-data-address-antimicrobial-resistance-kenya>  <https://www.reactgroup.org/news-and-views/news-and-opinions/year-2022/react-asia-pacific-antibiotic-smart-communities-as-a-way-forward/> | The SDG indicator 3.d.2 "Percentage of bloodstream infections due to selected antimicrobial-resistant organisms" is highly relevant for this Triple Billion indicator as antimicrobials include antibiotics. Therefore, the Global Partnership for Sustainable Development Data (GPSDD) and Africa’s Voices Foundation project that was implemented to generate and disseminate insights on AMR in Kenya through citizen participation is highly relevant for this indicator (see the 3.d.1 justification for further information).  Additionally, even though the project was designed for action, community engagement approaches such as the Antibiotic Smart Community Project involving communities and volunteers can help to understand patterns related to the antibiotic consumption in communities. The project was carried out in stages, including initial meetings with community leaders to raise awareness, a mapping of non-domestic antibiotic use sites using a Geographic Information System (GIS), and a community survey translated into the local language to gauge community awareness. Led by React, a global network that is active in five continents, the group has launched the project also in Pacific-Asia. | Direct |

**NOTES:**

For simplicity and to allow direct comparison with the 2020 systematic review by Fraisl et al., this table does not include the full review results from the 2020 review. For the full 2020 review results, see the Supplementary Material in Fraisl D, Campbell J, See L, et al (2020) Mapping citizen science contributions to the UN sustainable development goals. Sustain Sci 15:1735–1751. <https://doi.org/10.1007/s11625-020-00833-7>

The black font shows the results from the 2020 systematic review by Fraisl et al., while the blue font indicates the results of this particular study, which is focused on health and well-being related indicators.
